# Genome-wide expression analysis of vegetative organs during developmental and herbicide-induced whole plant senescence in *Arabidopsis thaliana*

**DOI:** 10.1186/s12864-024-10518-5

**Published:** 2024-06-19

**Authors:** Po-Yi Chen, Thi Thuy Tu Nguyen, Ruey-Hua Lee, Tsai-Wen Hsu, Ming-Hong Kao, Takashi Gojobori, Tzen-Yuh Chiang, Chao-Li Huang

**Affiliations:** 1 Department of Life Sciences, National Cheng Kung University, Tainan, 701 Taiwan; 2 Institute of Tropical Plant Sciences and Microbiology, National Cheng Kung University, Tainan, 701 Taiwan; 3 Taiwan Biodiversity Research Institute, Nantou, 552 Taiwan; 4 King Abdullah University of Science and Technology, 4700 KAUST Thuwal, 23955-6900 Kingdom of Saudi Arabia; 5 Graduate Program in Translational Agricultural Sciences, National Cheng Kung University and Academia Sinica, Tainan, 701 Taiwan

**Keywords:** *Arabidopsis thaliana*, Glyphosate, Nitrogen recycling, Phytohormones, RNA-seq, Senescence, Transcription factors, Transcriptional regulation

## Abstract

**Background:**

Whole plant senescence represents the final stage in the life cycle of annual plants, characterized by the decomposition of aging organs and transfer of nutrients to seeds, thereby ensuring the survival of next generation. However, the transcriptomic profile of vegetative organs during this death process remains to be fully elucidated, especially regarding the distinctions between natural programmed death and artificial sudden death induced by herbicide.

**Results:**

Differential genes expression analysis using RNA-seq in leaves and roots of *Arabidopsis thaliana* revealed that natural senescence commenced in leaves at 45–52 days after planting, followed by roots initiated at 52–60 days. Additionally, both organs exhibited similarities with artificially induced senescence by glyphosate. Transcription factors *Rap2.6L* and *WKRY75* appeared to serve as central mediators of regulatory changes during natural senescence, as indicated by co-expression networks. Furthermore, the upregulation of *RRTF1*, exclusively observed during natural death, suggested its role as a regulator of jasmonic acid and reactive oxygen species (ROS) responses, potentially triggering nitrogen recycling in leaves, such as the glutamate dehydrogenase (GDH) shunt. Root senescence was characterized by the activation of *AMT2;1* and *GLN1;3*, facilitating ammonium availability for root-to-shoot translocation, likely under the regulation of *PDF2.1*.

**Conclusions:**

Our study offers valuable insights into the transcriptomic interplay between phytohormones and ROS during whole plant senescence. We observed distinct regulatory networks governing nitrogen utilization in leaf and root senescence processes. Furthermore, the efficient allocation of energy from vegetative organs to seeds emerges as a critical determinant of population sustainability of annual *Arabidopsis*.

**Supplementary Information:**

The online version contains supplementary material available at 10.1186/s12864-024-10518-5.

## Background

Death signifies cessation of an individual’s life and biological functions, typically brought about by senescence in aged organisms. It is a natural and irreversible process that occurs in all organisms, with very few exceptions such as the immortal jellyfish [49]. In monocarpic plants, senescence represents a genetically programmed form of cell death, tightly regulated by complex pathways responsive to developmental and environmental cues [29, 35, 51]. Consequently, plants govern the timing, progression and completion of senescence as an essential strategy for reproduction, adaptation, fitness, and survival [7, 9, 66]. During the aging process, plants undergo changes in leaf morphology, source-sink balances, and chemical composition, including alterations in redox status and hormone levels [50, 54]. Senescence-related phytohormones, including jasmonic acid (JA), salicylic acid (SA), abscisic acid (ABA), and ethylene, play pivotal roles in promoting nutrient recycling and stress resistances in plant [10, 29, 39]. As plants undergo senescence preceding death, chlorophyll degradation occurs [70], leading to the withering of leaves [64]. Senescing leaves, serving as source organs, contribute nitrogen nutrient to sink organs via transport proteins such as NRT and AMT [48].

The timing of whole plant senescence is influenced by developmental age, which dictates how plants perceive and respond to environmental signals [47, 61]. Death often serves as a necessary step for survival, as plants allocate energy towards seed production before ultimately senescing to ensure survival of future generation. In some cases, plants enter the senescence phase as a consequence of reproductive growth cessation, when the plant ceases to produce new flower [21]. Additionally, environmental factors play crucial roles in determining the timing of reproductive senescence. These factors include the nutrient status of the plants [19], temperature [46], and day length [36]. Senescence and death in monocarpic plants are intricately coordinated with their flowering time under appropriate environmental cues, a crucial aspect for plant fitness and productivity [50]. The life cycle of *A. thaliana* begins with leaf senescence and culminates in death of siliques after seed maturation, ensuring the success of next generation. A study utilizing *Arabidopsis* plants of varying ages have demonstrated that senescence is synchronized and harmonized in response to environmental cues [47]. Leaf senescence follows a continuum pattern along the plant axis, occurring when leaves reach a certain age after completing their physiological tasks [28]. During senescence, nutrients are broken down and recycled to support the growth of young tissues, reproductive organs, or storage organs. Chloroplasts serves as the primary source of nitrogen recovery and reorganization in plants, contributing up to 75% of the nitrogen source [2, 22]. Leaf senescence represents the final phase of leaf development, regulated by the differential expression of thousands of genes at both genetic and epigenetic levels [7, 54, 58]. As plants progress towards death, roots play a critical role in supporting the final stage of life. Unlike seasonal dormancy, where roots primarily act as a storage organ to survive winters, senescing roots of annual plants tend to recycle the nutrients, particularly nitrogen (N), into seeds [33]. Given that nutrient acquisition is an energy-intensive process and nutrient recycling confers advantageous for plants [26, 53], the genomic expressions regulating programmed death, especially the senescence order of organs, are likely target of natural selection.

Numerous *Senescence-Associated Genes* (*SAGs*) have been identified in *Arabidopsis* [7, 9, 19, 23]. Using *Arabidopsis* as a model, approximately 10% of the total genes in the genome are up-regulated during senescence [54] with over 200 transcription factors implicated [8, 37, 61]. This suggested the involvement of a complex regulatory network in senescence. Achieving appropriate timing of senescence is crucial for plant productivity; however, the function of most *SAGs* in regulating senescence—particularly whole-plant senescence—remain largely elusive [46].

Developmental (natural) senescence occurs in plants grown under stress-free condition, where there is no presence of pests or diseases. In contrast, premature senescence may occur when plants encounter biotic and abiotic stresses, including extreme temperature, shading, drought, soil salinity, nutrient starvation, and exposure to toxic chemicals in the soil and air. Treating plants under continuous darkness is often used as experimental method to induce uniform senescence and compare the differential expression of *SAGs* with developmental senescence [35]. Endogenous factors such as age, reproductive development, and levels of hormonal regulators are important determinants of how plants respond to biotic and abiotic stresses, as well as the magnitude and severity of the stress, and the plant species involved [46]. Premature senescence is considering a protective mechanism that shortens the plant’s lifespan and to allocates valuable resources towards reproduction [4, 31]. This flexibility allow plants to respond to unfavorable environment and maximize their productivity [46, 61].

Glyphosate (N-phosphonomethyl glycine) is a broad-spectrum systemic herbicide used to control weeds and desiccate crop prior to harvest. Glyphosate treatment leads to growth inhibition, chlorosis, necrosis and subsequent plant death [13, 18]. When applied as a foliar spray, glyphosate is translocated to growing parts of the plant via vascular tissues. The systemic activity of glyphosate is critical for its effectiveness [15]. Glyphosate functions by inhibiting 5-enolpyruvylshikimate-3-phosphate synthase, a key enzyme in the shikimate pathway involved in the biosynthesis of tyrosine, tryptophan, and phenylalanine [40]. Growing shoots and roots are particularly susceptible to glyphosate due to their high rates of metabolism and growth [13, 52]. Glyphosate causes the accumulation of shikimate in plant tissues, redirecting energy and resources from other biosynthetic processes and ultimately leading to plant death [55]. Following glyphosate application, growth cessation occurs within hours, while leaf chlorosis typically manifests several days later [27].

The life cycle of annual *A. thaliana* typically spans approximately 6–8 weeks, encompassing stages of seed germination, vegetative growth, flowering, reproduction, aging and eventual death. Rosette leaves, originating from the primary shoot meristem, undergo transformation into floral meristem approximately three weeks after germination [12]. At the reproductive stage, the plant typically develops six to 10 rosette leaves. Once the decision to flower is made, there is no reversal; the plant proceeds to set seeds and undergoes senescence [62]. Three distinct stages of the aging and death process of *A. thaliana* have been identified [69]. The first stage occurs from 31^st^ to 39^th^ day since germination, characterized by the rosette leaves turning light green due to chlorophyll degradation. Subsequently, from 39^th^ to 52^nd^ day, the rosette leaves transition from light green to yellow, accompanies by high rate of chlorophyll degradation and reduced photosynthesis efficiency. During this stage, *A. thaliana* reallocates energy from degraded chlorophyll to the reproductive organs to compensate for the imbalance in energy supply. The third stage occurs between the 52^nd^ and 60^th^ day, during which the leaves wilt, and the seeds mature. At this point, the plant mobilizes nutrients from the roots to nurture the ripening seeds. Ultimately, as root senescence progresses, the plants experience a decline in water and nutrition supply, leading to death.

Miryeganeh et al. [47] conducted gene expression analysis using the least senescent leaves of *A. thaliana* and observed the upregulation of SAGs 2–4 weeks prior to whole plant senescence. Notably, senescence-responsive genes such as *SEN4* and *SAG12* have been identified as key regulators in detecting senescence [16]. While the molecular mechanism of leaf senescence and physiological response during flowering have been extensively studied in *Arabidopsis* [56], the molecular basis of root senescence and whole plant senescence remains to be fully elucidated.

In this study, we employed high-throughput RNA sequencing to investigate dynamic transcriptome changes in *A. thaliana* plants undergoing natural senescence and senescence induced by the herbicide glyphosate. Leaves and roots of *A. thaliana* were harvested at three time points: the 45^th^, 52^nd^, and 60^th^ day after seed sowing, to examine gene expression during natural senescence. The period from the 45^th^ to 52^nd^ day was designated as the first stage of natural death (ND1), while the interval from the 52^nd^ to 60th day was labeled as the second stage of natural death (ND2). Harvesting occurred prior to complete plant death. By analyzing the dynamic transcriptomic profiles during senescence under both natural and herbicide-induced conditions, we aim to gain insights into how plant respond to the most widely used herbicide worldwide. The study seeks to address several key questions: (1)What are the gene expression profiles of leaves and roots during the whole plant senescence of *A. thaliana*? (2)What mechanisms underlie the triggering of the programmed and artificial death of *A. thaliana*? (3)How do transcription factors participate in the transfer of energy during both programmed and artificial death?

## Methods

### Plant materials

*Arabidopsis thaliana* ecotype Columbia (Col-0) was selected as an experimental model to investigate genomic expression preceding senescent death in plants. Seeds of *Arabidopsis* were generously provided by Dr. Munetaka Sugiyama and formally identified by Dr. Tsai-Wen Hsu (Voucher ID: 24,370 in the TAIE herbarium). All local, national or international guidelines and legislation were adhered to in the production of this study. Both natural and artificially induced senescent deaths of *Arabidopsis* were examined. Seeds were soaked in water and cold-stratified at 4℃ in darkness for five days. Vernalized seeds were then sown on a compost mix containing of Jiffy substrate and vermiculite at ratio of 6:1 in plug trays. Each cell contained three seeds, and the tray was covered with plastic wrap before being placed in a growth chamber under a 16-hour light/8-hour dark cycle at 22℃. Upon the development of four rosette leaves (approximately 10–15 days), only one plant per cell was retained for further experimentation.

For the natural senescence experiment, rosette leaves and roots were collected from *Arabidopsis* plants aged 45 days (for 50% flowering according to Boyes et al. [6] marked by the onset of light green coloration in rosette leaves ), 52 days (when siliques start maturing) and 60 days (when rosette leaves start turning yellow). Samples were collected in two biological replicates (see Supplementary Table S1 for details). For the artificial senescence experiment, 29-day-old plants were sprayed with a 100X diluted solution of glyphosate (Yih Fong chemical corp., Taiwan) or distilled water (see Supplementary Table S2). Each plant was sprayed three times from a distance of 15 cm above the rosette leaves. Glyphosate-treated plants were kept in the growth chamber for an additional two days before rosette leaves and roots were collected in three biological replicates. Compost on the root samples was removed by rinsing with distilled water. Both leaf and root samples were immediately frozen in liquid nitrogen and stored at -80 °C for total RNA isolation.

### Total RNA isolation, quality check and sequencing

Total RNA isolation was conducted using a modified cethyl-trimethylammonium bromide (CTAB)-based extraction method [3]. The extraction buffer consisted of 2% (w/v) CTAB, 100 mM Tris–HCl (pH 8.0), 2 M NaCl, 25 mM EDTA (pH 8.0), and 0.05% (w/v) spermidine. Prior to use, polyvinylpyrrolidone K30 (2%), β-mercaptoethanol (2%), and proteinase K (10 mg/ml) were added to the RNA extraction buffer, which was then incubated at 42℃. Plant tissues were ground into a fine powder using liquid nitrogen, and 5–10 volumes of preheated extraction buffer were added. The sample mixes were incubated at 42℃ for 90 min with vortexing at 10 min intervals. Tissue mixtures were then extracted at least twice with an equal volume of chloroform: isoamyl alcohol (24:1) until the interface was clear after centrifugation at 15,000×g at 4 °C for 15 min. The supernatant was transferred to a fresh tube, and 0.25 volume of 10 M lithium chloride (LiCl) was added. The mixture was left overnight at 4 °C for RNA precipitation. RNA was pelleted by centrifugation at 15,000×g at 4℃ for 25 min, and the supernatant was discarded. The pellet was washed with 2 M LiCl and centrifuge at 15,000×g at 4ºC for 25 min. The air-dried pellet was dissolved in 100–200 µl DEPC-treated water and stored at -80℃ until further use. RNA concentration was measured by Qubit BR RNA kit, and samples with concentration greater than 80 ng/µl were selected. All RNA samples were sequenced using the HiSeq 2500 platform (Illumina) with a 50-bp single-end sequencing (Yourgene Bioscience Corp., Taiwan).

### Bioinformatics analysis

RNA-seq was employed to compare the genome-wide expression profiles in leaves and roots between naturally and artificially induced senescent conditions. Raw reads were aligned to the *A. thaliana* TAIR10 reference genome using Bowtie2 (ver. 2.3.0) and Tophat2 (ver. 2.0.14) with default parameters. The aligned reads were assembled using the Cufflinks package (ver. 2.2.1) with the reference annotation-based transcript (RABT) assembly method. The Cuffmerge command was utilized to merge transcript assemblies, resulting in a single set of predicted transcripts. The merged GTF files were then employed in differential expression analysis using Cuffdiff. Expression levels of different transcripts were calculated based on abundances from aligned reads for each sample, followed by statistical analysis. Transcript counts with less than 10 counts were excluded to ensure robustness. We examined the expression level of three senescence marker genes: *Cab3*, *SAG12* and *SEN4*. Using day 39 as the baseline, we analyzed the fold change in expression levels at days 45, 52 and 60. The results indicated that the expression of Cab3 increased earlier at day 45, while the expression of SAG12 and SEN4 increased at day 52. This data suggested that the onset of leaf senescence occurred between day 45 and day 52 in this experiment [5]; Supplementary Fig. S1). Differentially expressed genes was conducted for the following sample comparisons: (i) LND1: 45-day-old leaf vs. 52-day-old leaf; (ii) LND2: 52-day-old leaf vs. 60-day-old leaf; (iii) 45-day-old leaf vs. 60-day-old leaf ; (iv) RND1: 45-day-old root vs. 52-day-old root; (v) RND2: 52-day-old root vs. 60-day-old root; (vi) 45-day-old root vs. 60-day-old root; (vii) LAD: glyphosate-treated leaf vs. control leaf; and (viii) RAD: glyphosate-treated root vs. control root.

The coefficient of variation of FPKM (Fragments Per Kilobase per Million) of genes in leaves and roots undergoing natural and artificial death were calculated and visualized using histograms generated by the “hist” function in R Studio. False Discovery Rate (FDR) correction was applied to account for multiple testing correlations [60]. Genes displaying a logarithm base 2 of fold change greater than 0 are categorized as up-regulated genes, while those with values less than 0 were considered down-regulated genes. Differential expression between samples was determined based on a fold change of ≥ 2 and an FDR-corrected *P* value < 0.05. Venn diagrams illustrating the overlap differentially expressed genes (DEGs) in leaves and roots were created using the web tool “Calculate and draw custom Venn diagrams (http://bioinformatics.psb.ugent.be/webtools/Venn/)”.

Transcription factors were identified among the up-regulated DEGs using PlantPAN 3.0 [11], employing the following parameters: 1,000 base pairs upstream and 100 base pairs downstream of the transcription start site, and 500 base pairs downstream of the transcription termination site. Gene functional enrichment analysis was performed using DAVID (The Database for Annotation, Visualization, and Integrated Discovery) version 6.8, incorporating gene ontology (GO) and the KEGG pathway annotations. A GO term was deemed enriched if the Bonferroni-corrected *P* value was < 0.05. Heatmaps of DEGs and enriched GO terms were generated using MeV 4.9.0 with Z score based on the logarithm base 2 of the Bonferroni-corrected *P* value. Co-expression analysis was conducted using EXPath 2.0 [63] with Spearman’s rank correlation coefficient (Spearman’s rho) and a cut-off value of 0.8. The resulting co-expression network was visualized using Gephi 0.9.2.

## Results

### Overview of RNA-seq results

In this study, samples were collected in duplicate for natural senescence and triplicate for glyphosate-induced senescence (Supplementary Table S3). Illumina sequencing revealed consistent read counts across different sampling days for both leaves and roots. For leaves, alignment rates ranged from 95.8 to 96.6%, while for roots, alignment rates ranged from 90.3 to 94.5%. In the glyphosate-treated experiment, alignment rates were consistently high, ranging from 98.0 to 98.5% for leaves and from 97.6 to 98.1% for roots. The numbers of detected genes varied slightly across sampling days and treatments, with leaf samples detecting 20,355 to 21,107 genes in the natural senescence experiment and 33,019 to 33,202 genes in the glyphosate-treated experiment (Supplementary Table S4). Similarly, root samples detected 21,604 to 22,068 genes in the natural senescence experiment and 33,019 to 33,267 genes in the glyphosate-treated experiment (Supplementary Table S4).

### The differential expression analysis indicates leaf senescence occurring earlier than root senescence

**Fig. 1:**
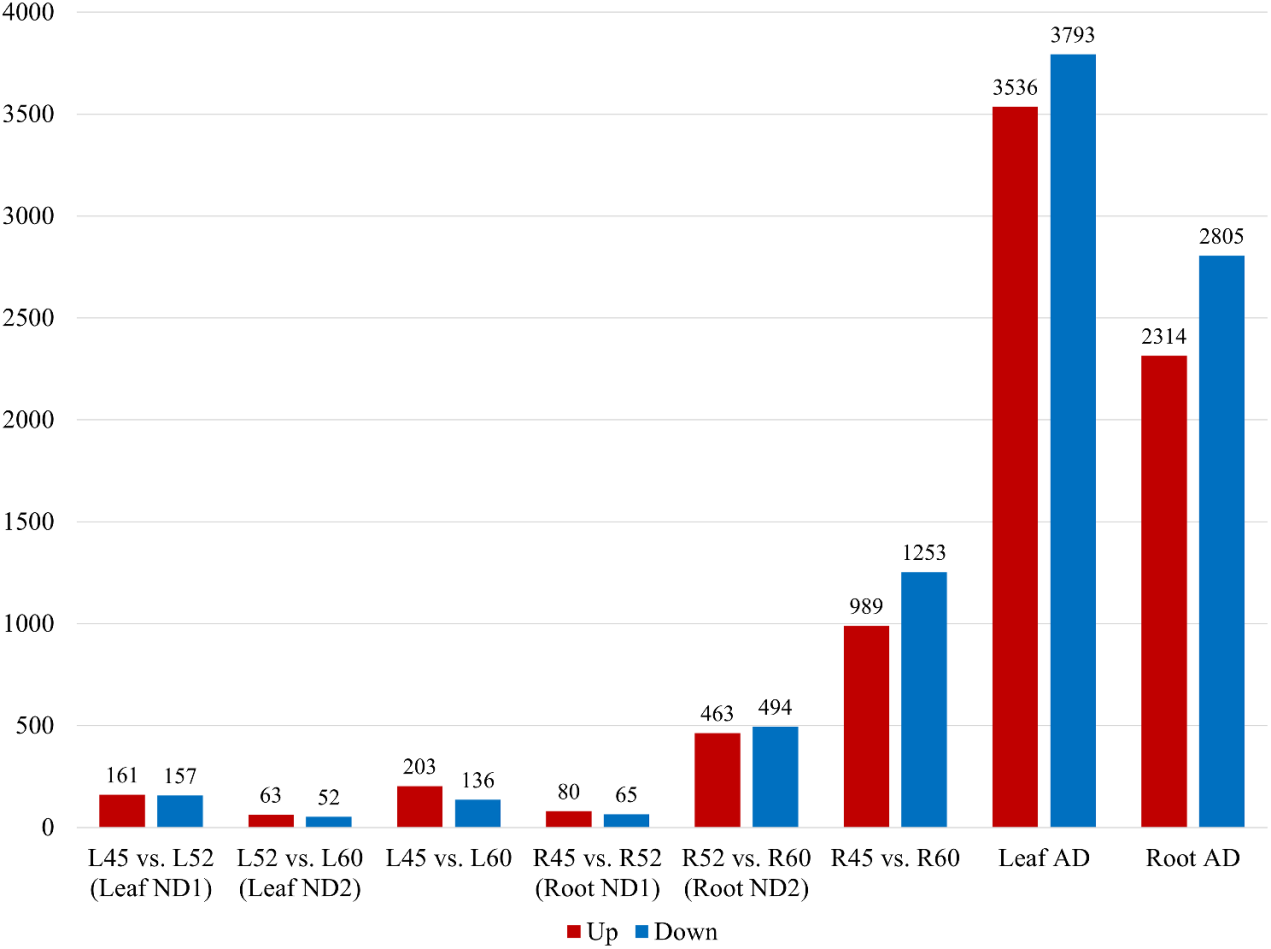
The number of differentially expressed genes (DEGs) in leaves and roots under natural and artificial death conditions. The ordinate and abscissa indicate the number of DEGs and tissues and stages, respectively. The red bar means up-regulated DEGs, and the blue bar means down-regulated DEGs

Differentially expressed genes (DEGs) were identified from the RNA-seq data using Cuffdiff (Fig. 1; see supplementary S5 for details). In total, 318 DEGs were detected in the 1^st^ stage of leaf natural senescence (LND1), comprising 161 up-regulated genes and 157 down-regulated genes. Similarly, 115 DEGs were identified in the 2^nd^ stage of leaf natural senescence (LND2), with 63 up-regulated genes and 52 down-regulated genes. In addition, 339 DEGs were detected in the comparison across the two stages (45-day-old vs. 60-day-old leaves), consisting of 203 up-regulated and 136 down-regulated genes. In roots, 145 DEGs were found in the 1^st^ stage of natural senescence (RND1), with 80 genes showing increased expression and 65 genes showing decreased expression. Additionally, 957 DEGs were identified in the 2^nd^ stage of natural senescence in roots (RND2), consisting of 463 genes with increased expression and 494 genes with decreased. Moreover, 2,242 DEGs were detected in the comparison across the two stages (45-day-old vs. 60-day-old roots), comprising 989 up-regulated and 1,253 down-regulated genes. In the case of glyphosate induced senescence, 7,329 DEGs were detected in leaves (LAD), with 3,536 genes showing up-regulation and 3,793 genes showing down-regulation. Similarly, 5,119 DEGs were identified in roots (RAD), comprising 2,314 up-regulated genes and 2,805 down-regulated genes.

#### Venn diagram of DEGs shows that gene regulation between LND1 and LAD, as well as RND2 and RAD, shares high similarities

**Fig. 2:**
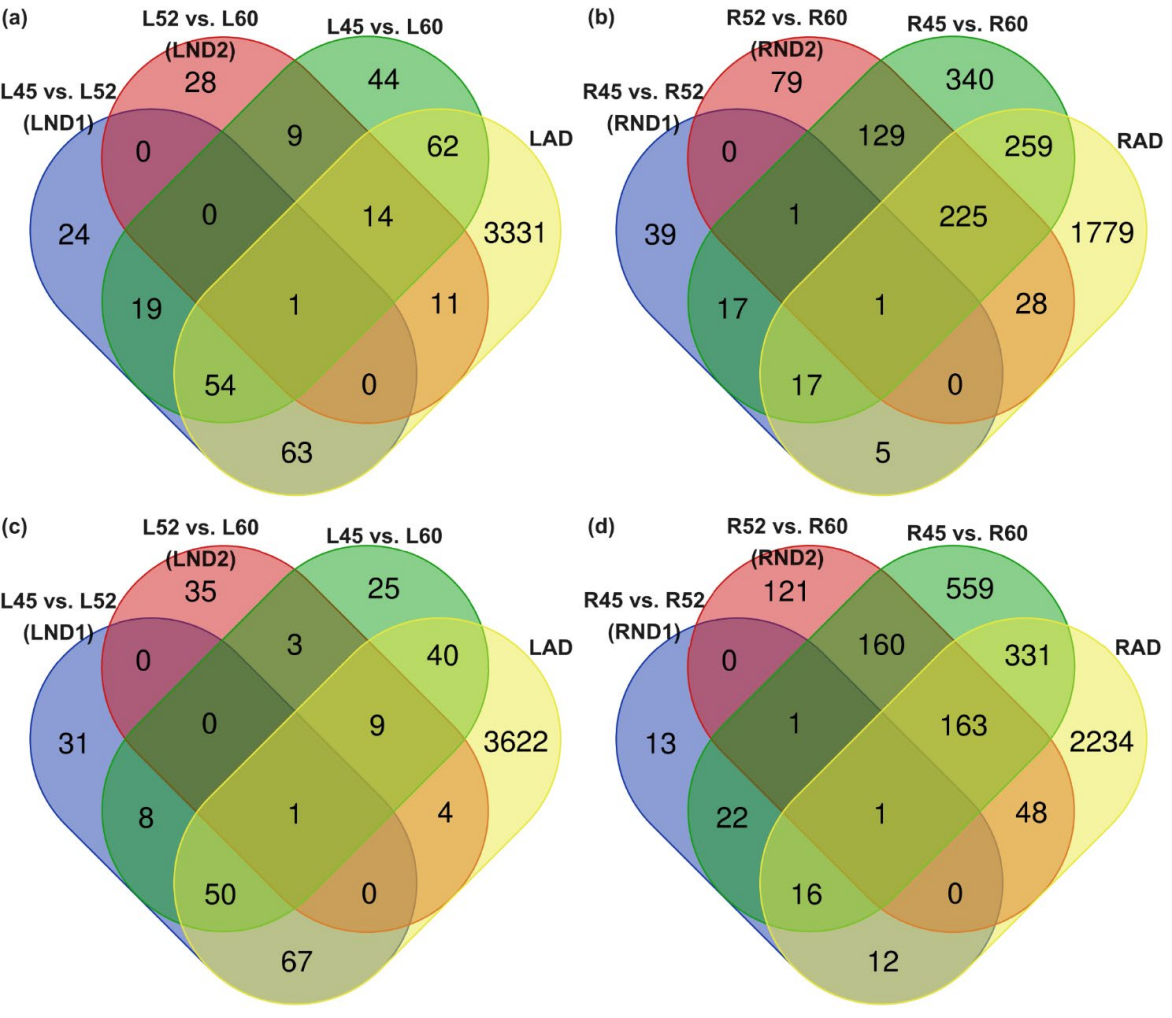
The Venn diagram indicating differentially expressed genes. Up-regulated genes in (**a**) leaves and (**b**) roots under natural and artificial death conditions, as well as down-regulated genes in (**c**) leaves and (**d**) roots

In leaves, the Venn diagram revealed 24 up-regulated DEGs in LND1, 28 in LND2, 44 in the comparison of 45-day-old vs. 60-day-old, and 3,331 exclusively in LAD (Fig. 2). There were 118 up-regulated DEGs shared between LND1 and LAD, and 26 shared between LND2 and LAD. Among all these shared DEGs, only one up-regulated gene was common to all leaf comparisons. Regarding down-regulated DEGs, 31 genes were exclusively detected in LND1, 35 in LND2, 25 in the comparison of 45-day-old vs. 60-day-old, and 3,622 in LAD. There were 118 genes shared between LND1 and LAD, and 14 between LND2 and LAD. Among all these shared DEGs, only one down-regulated gene was common to all leaf comparisons. In roots, 39 up-regulated DEGs were exclusively detected in RND1, 79 in RND2, 340 in the comparison of 45-day-old vs. 60-day-old, and 1,779 up-regulated DEGs were exclusively detected in RAD. There were 23 genes shared between RND1 and RAD, and 254 shared between RND2 and RAD. Similarly, 13 down-regulated DEGs exclusively occurred in RND1, 121 in RND2, 559 in the comparison between 45-day-old and 60-day-old roots, and 2,234 in RAD. There were two down-regulated DEGs shared between RND1 and RND2, 29 shared between RND1 and RAD, and 212 shared between RND2 and RAD. Again, among all these shared genes, only one up-regulated DEG and one down-regulated DEG were common to all root comparisons. Comparing the numbers of up-regulated and down-regulated DEGs, more shared DEGs were found between LND1 and LAD, and between RND2 and RAD. These results suggest that the gene regulation of LND1 and LAD, as well as those of RND2 and RAD, shared relatively high similarities.

#### The heatmap of enriched biological process and transcription factors revealed the role of phytohormones in regulating whole plant senescence in Arabidopsis

**Fig. 3:**
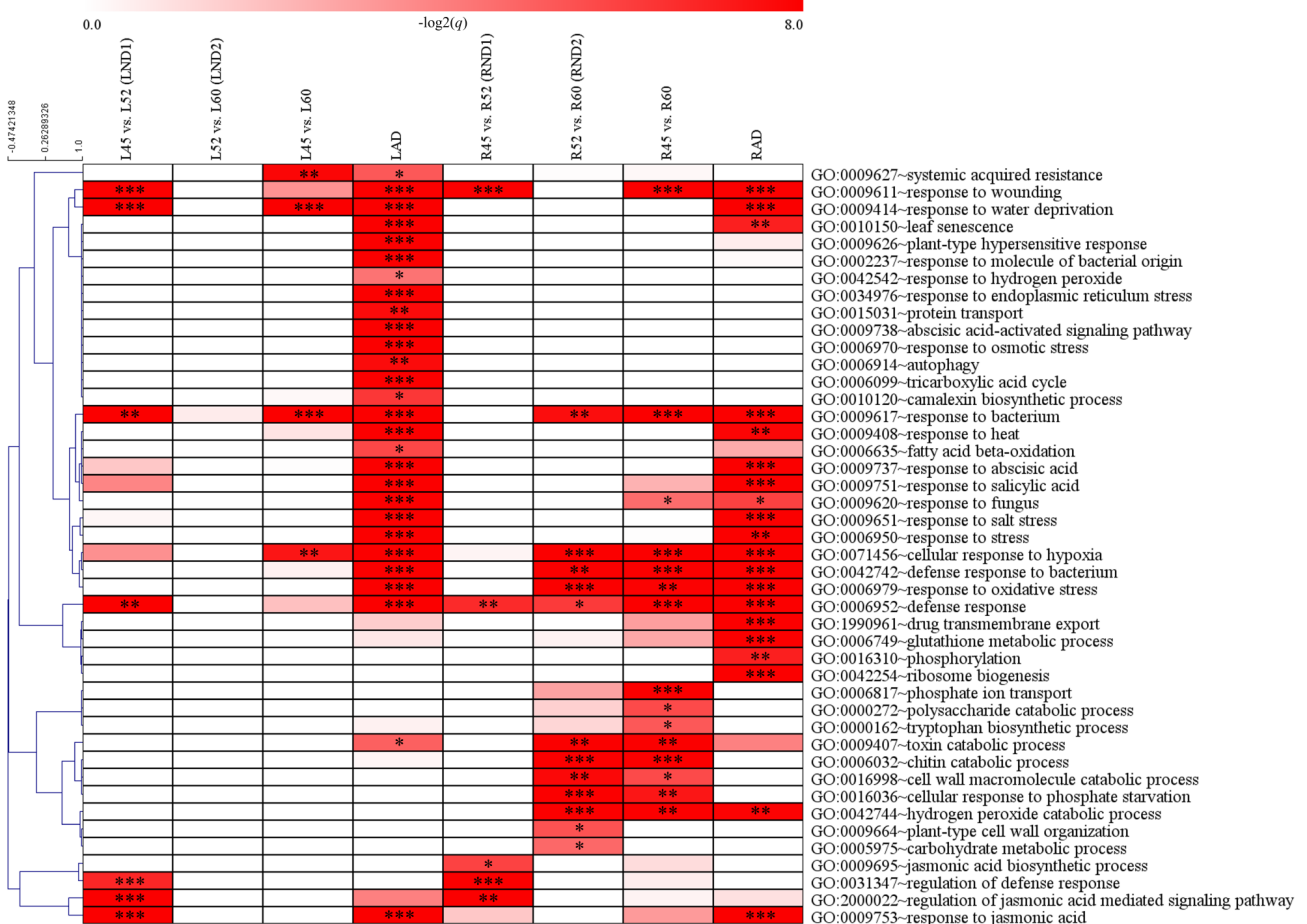
The heatmap of biological process in leaves and roots under natural and artificial death conditions. Heatmap was color-coded as per the Bonferroni-corrected value of biological process (-log_2_(*q* value)). Complete-linkage clustering was used via Pearson correlation. The significant *q* value was presented in each cell (*q < 0.05; ** *q* < 0.01; *** *q* < 0.001)

In LND1, 161 up-regulated DEGs were associated with seven enriched biological processes, five of which were related to stresses, and two involved the response to the phytohormone jasmonic acid (GO:0009753 and GO:2,000,022) (Fig. 3). Surprisingly, among the 63 up-regulated DEGs in LND2, no enriched process was identified. Conversely, in the case of artificially induced senescence, 28 enriched processes were detected, including two related to oxidative processes, 12 related to biotic and abiotic stresses, and the process of leaf senescence (GO:0010150). Four enriched biological processes were identified by comparing 45-day-old and 60-day-old leaves, including systemic acquired resistance (GO:0009627) and the cellular response to hypoxia (GO:0071456), which were detected exclusively. Most of up-regulated processes in LND1 were not shared with the comparison of 45-day-old vs. 60-day-old, indicating these processes were only detected in a specific period. The heatmap revealed that the enriched biological processes in LND1 and LAD were quite similar with all five processes enriched in both LND1 and LAD being related to stress responses. Regarding down-regulated DEGs, the biological process related to auxin response was enriched in LND1, while those involved in photosynthesis were enriched in LAD (Supplementary Tables S6–S8).

In the RND1, several responses to stress and two GO-terms of JA process, namely jasmonic acid biosynthetic process (GO:0009695) and regulation of jasmonic acid mediated signaling pathway (GO:2,000,022), were detected. Notably, JA-related processes were also enriched in LND1 (response to jasmonic acid, GO:0009753), suggesting that JA plays a significant role in regulating the early senescence stage of both leaves and roots. In RND2, we identified 12 enriched biological processes, including various macromolecule metabolisms processes, such as those related to cell wall, carbon, nitrogen, and sulfur metabolisms. Fifteen biological processes were enriched in the comparison of 45-day-old vs. 60-day-old, most of which were shared with RND2. However, four biological processes were exclusively enriched in the comparison of 45-day-old vs. 60-day-old, indicating some functions continuously up-regulated throughout the senescence stages in roots, including phosphate ion transport (GO:0006817), polysaccharide catabolism (GO:0000272), and tryptophan biosynthesis (GO:0000162). In RAD, 20 biological processes were enriched, including responses to phytohormones such as jasmonic acid (GO:0009753), abscisic acid (GO:0009737), salicylic acid (GO:0009751), and ethylene (GO:0009723 and GO:0009873), as well as responses to biotic and abiotic stresses. The analysis revealed differences in the enriched biological processes between RND2 and RAD, with macromolecule metabolic processes predominating in RND2 and phytohormonal processes being more enriched in RAD compared to RND1 and RND2. However, responses to bacteria and redox were enriched in both death processes. Regarding the down-regulated DEGs, responses related to cell wall biosynthesis were detected in RND2 and RAD (Supplementary Tables S9–S11).

**Fig. 4:**
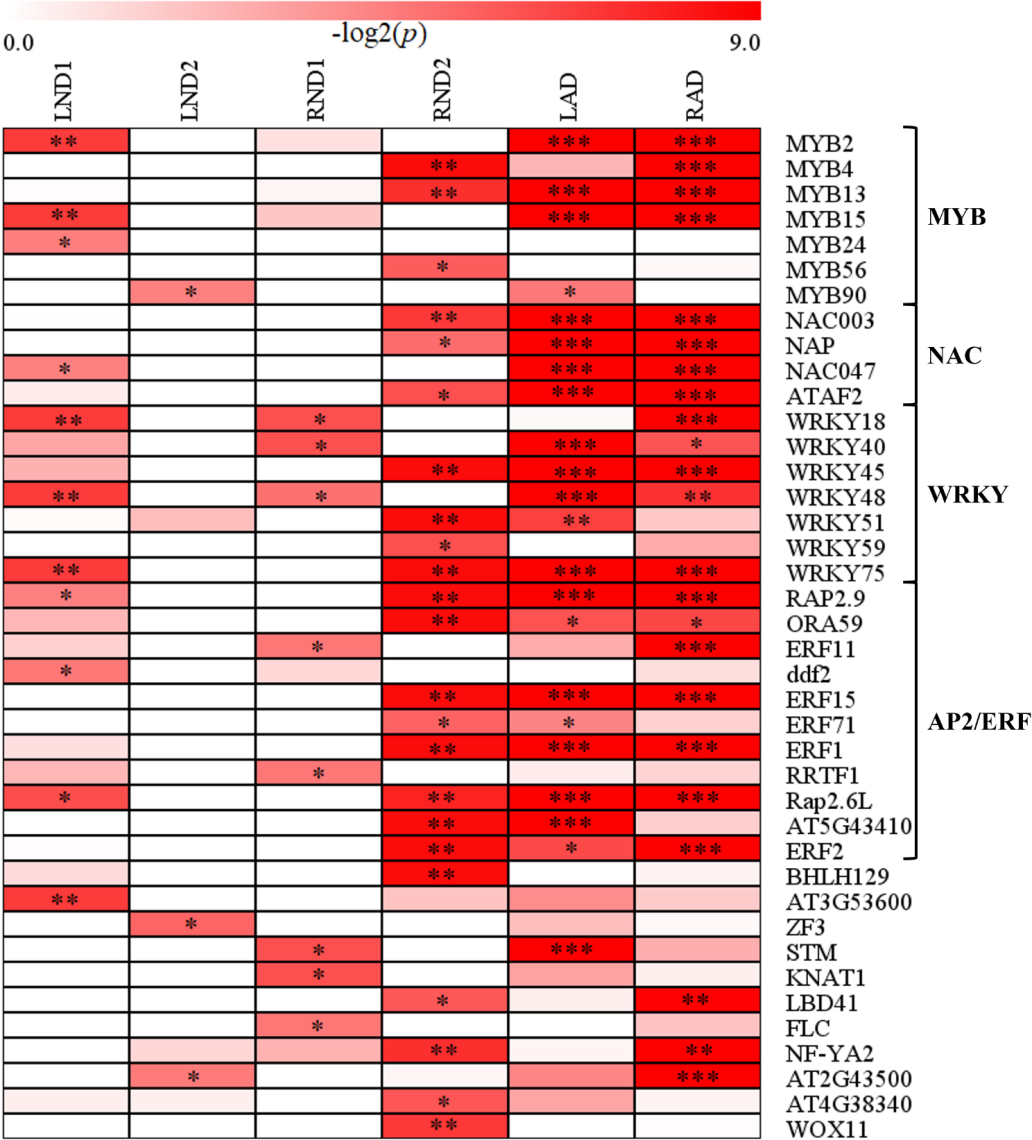
The heatmap of differential expression of transcription factors in leaves and roots under natural and artificial death conditions. The Bonferroni-corrected *q* value of transcription factor was transferred by -log_2_. The significant *q* value was presented in each cell (*q < 0.05; ** *q* < 0.01; *** *q* < 0.001)

In both leaves and roots undergoing natural senescence, a total of 40 transcription factors (TFs) were enriched, representing four families of TFs: *MYB*, *NAC*, *WRKY* and *ERF* (Fig. 4). Specifically, in LND1, 11 TFs were enriched, including *MYB2*, *MYB15*, *MYB24*, *NAC047*, *WRKY18*, *WRKY48*, *WRKY59*, *WRKY75* and *Rap2.6L*. In contrast, only three TFs, such as *MYB90*, were enriched in LND2. In roots, eight TFs were enriched in RND1, including *WKRY18*, *WKRY40*, *WRKY48*, *ERF11*, *FLC* and *STM* (*Shoot Meristemless*). In RND2, 23 TFs were enriched in RND2, including *MYB4*, *MYB13*, *MYB56*, *NAC003*, *NAP*, *WRKY45*, *WRKY51*, *WRKY59*, *WRKY75*, *Rap2.6L* and *ERF1*, *ERF2*, *ERF15*, *ERF71*.

### Co-expression network of DEGs

**Fig. 5:**
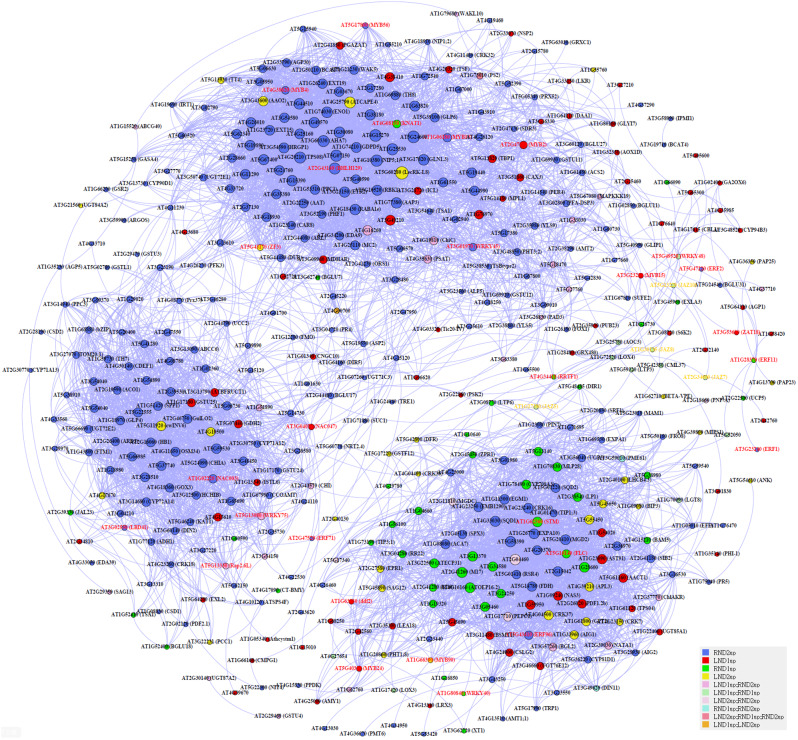
The co-expression network of up-regulated differentially expressed genes in leaves and roots under the 1st stage (ND1) and the 2nd stage (ND2) of natural death. Co-expressed genes were provided based on Spearman’s rank correlation coefficient via EXPath 2.0. The red letter means transcription factor genes and the yellow letter means jasmonate-zim domain genes

The co-expression network analysis revealed a total of 458 nodes and 6,868 edges, with 26 TFs identified (Fig. 5). Notably, the network displayed three distinct groups, each characterized by specific hub nodes and co-expression patterns. In one group, TFs *FLC* and *STM* emerged as central hubs, orchestrating a network of interactions. *SAG12* exhibited co-expression with *STM*, *NRT2.4* (*Nitrate Transporter 2.4*) and *AMY1* (*Amylase Alpha 1*). Additionally, *JAZ5/7/8/10* formed a tightly interconnected sub-network, with *JAZ5* co-expressed with *LOX4* (*lipoxygenase 4*), while *JAZ7* and *JAZ10* co-expressed with TF *RRTF1* (*Redox Responsive Transcription Factor 1*) (Supplementary Table S12). Another notable finding was the presence of *WRKY75*, identified as a TF enriched in both leaves and roots across natural and artificial senescence conditions. *WRKY75* exhibited co-expression with *NAC003*, *Rap2.6L* and *GDH2*, serving as central nodes within a distinct subgroup associated with organ senescence processes. Moreover, *MYB2* emerged as a central hub within the network, demonstrating co-expression with *AMT2* (*Ammonium Transporter 2*) and *GLN1.3* (*Glutamine Synthetase 1.3*). This observation suggested a potential role for *MYB2* in regulating nitrogen utilization pathways.

#### Coefficient of variation of gene expression in leaves and roots of natural and artificial death

**Fig. 6:**
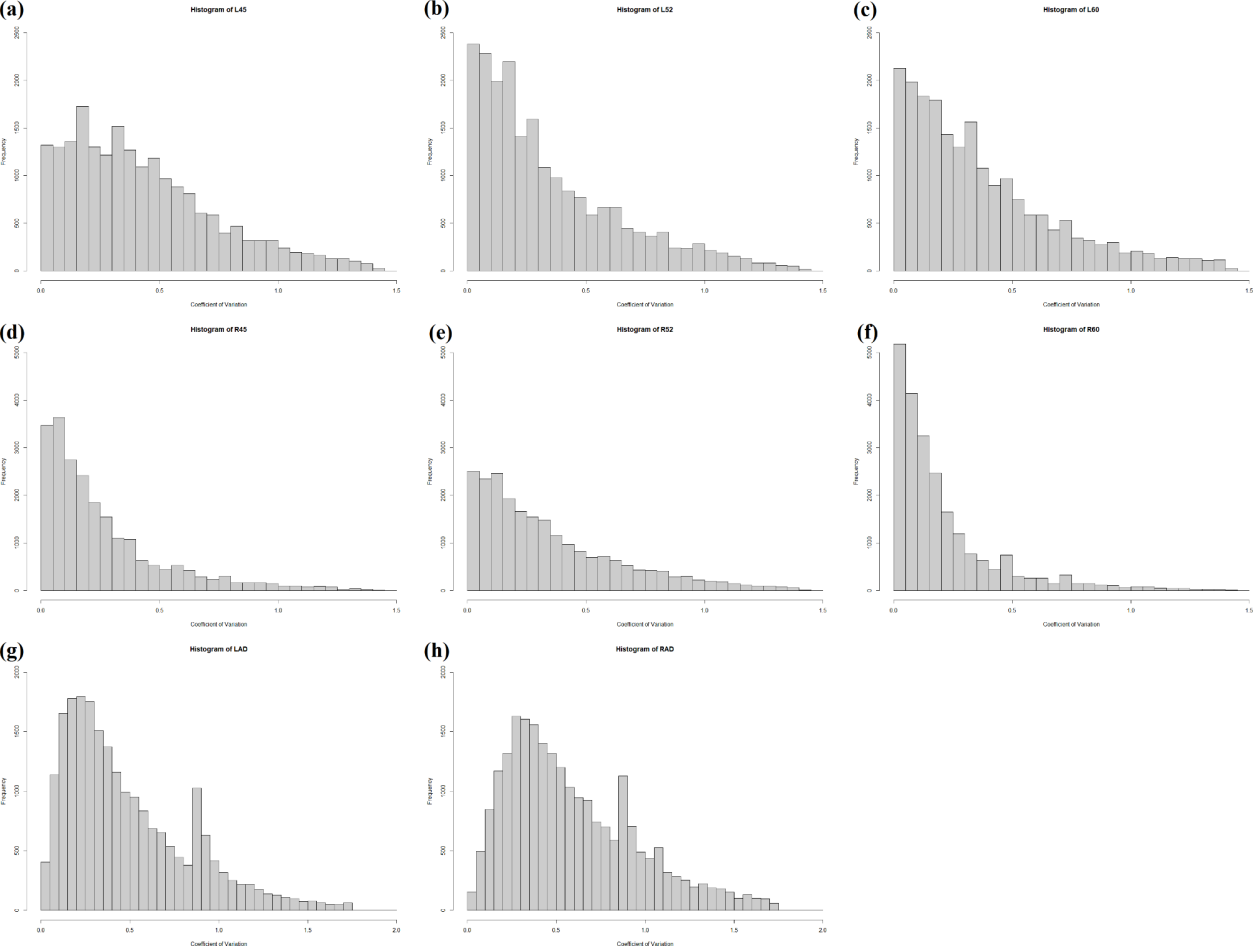
Histogram of coefficient of variation (CV) of gene expression in different day of natural death (ND) and artificial death (AD). Genes occurring in one sample only were removed. (**a**) The 45^th^ day of gene expression in leaf ND. (**b**) The 52^nd^ day of gene expression in leaf ND. (**c**) The 60^th^ day of gene expression in leaf ND. (**d**) The 45^th^ day of gene expression in root ND. (**e**) The 52^nd^ day of gene expression in root ND. (**f**) The 60^th^ day of gene expression in root ND. (**g**) The gene expression in leaf AD. (**h**) The gene expression in root AD

A total of 24,421 genes were detected in LAD, while 25,563 genes were detected in RAD. To elucidate the distribution of genes expression among samples, we examined the patterns in coefficient of variation (CV) (Fig. 6G, H). Among the genes, 2,280 (9.4%) in LAD and 2,364 (9.2%) in RAD exhibited considerable scattering across samples. Notably, with CV values less than 0.7, a substantial proportion of genes—16,687 (68.3%) in LAD and 15,608 (61.1%) genes in RAD—were consistently expressed across all samples. We designated these as “core” senescence genes, representing robust and stable expression patterns through the experimental conditions. Interestingly, approximately 31.7–38.9% of expressing genes displayed sporadic distribution across samples, suggesting unpredictable responses within the entire genome. In contrast, a higher percentage of genes exhibited stable expression patterns in the context of natural senescence (ranging from 65.9 to 90.2% of genes with CV values less than 0.5) (Fig. 6A-F).

## Discussion

### Gene regulation network in natural death of leaves and roots

During the natural death of an annual plant, leaf senescence typically precedes overall senescence, including root senescence [14]. While gene regulations in *A. thaliana* have been extensively studied in detail, the interplay between the two organs for ensuring a “successful” death process remains incompletely understood. Our analysis of the DEGs indicated that leaf senescence occurs between 45 and 52 days after planting (DAP), while root senescence initiated around 52–60 DAP (Fig. 2). Notably, the well-known aging factor *SAG13* was activated during senescence (Supplementary Table S13), indicating its involvement in the process. Additionally, the up-regulation of *SAG12* occurred later (Supplementary Table S13), specifically in the final stage of leaf death, suggesting a potential role in the “stay-green” hypothesis. This hypothesis posits that prolonged longevity of leaves supports seed development [34].

Our data revealed a more vigorous and complex transcriptomic transition during root senescence compared to leaf senescence, indicating a more precise regulation in the latest stage of the death process. Notably, pathways involving reactive oxygen species (ROS) were particularly prominent in these regulatory changes. In RND2, several peroxidase-encoding genes, such as AT1G71695 and AT2G37130 (Supplementary Table S14), were up-regulated. These genes are primarily involved in plant-type cell wall organization, suggesting that the integrity of cell wall may play a crucial role in the natural death of roots. In RND2, the up-regulation of *PDF2.1*, which encodes a functional peptide located on cell wall, suggests a potential role regulating genes involved in nitrogen transport, including *AMT2;1* and *GLN1;3* [67] (Supplementary Table S13). *AMT2;1* facilitates the root-to-shoot translocation of ammonium, contributing to the nutrition recycling of senescing roots [20], while *GLN1;3* encodes a glutamate synthetase involved in assimilating free ammonium in plant cells [32]. Both genes were significantly up-regulated at RND2. Therefore, the highly expressed *PDF2.1* may collaborate with *AMT2;1* and *GLN1;3* to scavenge the ammonium released during root death, with the root-to-shoot translocation possibly require intact cell wall integrity for efficient nutrient transport.

Our study revealed JA initiates early signals of natural death in both leaves and roots. A JA-related TF, *RRTF1* co-expressed with JA transcriptional repressors, JAZ7 and JAZ10 (Fig. 5), integrating ethylene and auxin-activated signaling pathway for organ regeneration [68]. *RRTF1* is induced by JA and stimulates ROS accumulation under stress conditions [42]. As RRTF1 was up-regulated prior to the mass expression changes in roots, its role might be involved in initiation ROS for triggering root death of *A. thaliana*. In addition, *RRTF1* is co-regulated with another JA-inducible TF, *Rap2.6* (AT1G43160), as both contain a GCC-box-like motif in the promotor region [42]. One paralogs of *Rap2.6*, *Rap2.6L* (AT5G13330), induced by JA and ABA [38, 43], was up-regulated in the key stages of natural death in leaves and roots of *Arabidopsis* (LND1 and RND2) (Supplementary Table S13). Increased expression of *Rap2.6L* also induces *ABA1*, which functions in ABA biosynthesis, suggesting that the endogenous ABA level could be regulated by Rap2.6L [38]. Since Song et al. [59] indicate endogenous ABA level is a key factor in initiating leaf senescence, the earlier up-regulation of *Rap2.6L* in leaves compared to roots likely led to earlier leaf senescence by mediating ABA signaling pathway. Furthermore, *MYB2*, induced in leaf senescence and co-expressed with *AMT2;1* and *GLN1.3*, is also involved in the ABA signaling [1] (Fig. 5; Supplementary Table S13). Hence, the roles of *AMT2;1* and *GLN1.3* in root death may also be regulated via *MYB2* and ABA signaling.

Moreover, the TF *WRKY75*, co-expressed with *Rap2.6L* (Fig. 5), is known to regulate downstream *SID2* and *CAT2*, thereby promote SA biosynthesis ROS generation [24]. *WRKY75* was consistently induced in leaves and roots in both natural and artificial deaths (Supplementary Table S13), suggesting its central role in promoting general death processes [65]. The co-expression network of up-regulated DEGs identified 39 genes associated with *WRKY75* (Fig. 5). Among them, the gene encoding glutamate dehydrogenase 2 (GDH2) was activated in LND1 (Fig. 5; Supplementary Table S13). *GDH2* is known as a marker gene for leaf senescence [25]. As the plant initiated death process, the efficiency of carbon fixation is reduced due to the degradation of photosynthetic apparatus, leading to a carbon-limited state. Additionally, leaves mobilize the nitrogen released from chloroplast degradation, resulting in elevated level of ammonia causes local C/N imbalances, which require assistance from GDH, known as the GDH shunt [45]. With GDH activity, glutamate is degraded to ammonia and 2-oxoglutarate, which could be utilized to synthesize glutamine for nitrogen transportation. For an annual plant such as *A. thaliana* at end-of-life stage, increasing *GDH2* expression may maximize the nutrient acquisition for seed development would be a key for species sustainability, suggesting the importance of maintaining C/N balance at the first stage of natural death. These changes delivered a death signal and thus influenced the transcriptomic transition in senescing roots.

Another TF *ERF2* was enriched in RND2 and co-expressed with *LOX4* (Fig. 5; Supplementary Table S13). *ERF2* was involved in the signaling pathway of ethylene and JA [17, 44]. In contrast to the predominant roles of JA in natural death of leaves, TFs of the ERF family were preferentially enriched in RND2, suggesting that ethylene may primarily contribute to the natural death of roots (Fig. 4; Supplementary Table S13). Besides, NAP, a TF of NAC family promoted by ethylene [30] and up-regulated during leaf lifespan [65], was enriched in RND2, further supporting the assumption (Fig. 4).

### Natural death vs. glyphosate-induced artificial death

Previous studies have indicated that processes related to disassembly are predominantly up-regulated during the leaf lifespan. Specifically, catabolic processes of chlorophylls and proteins are particularly prominent in the later growth stage [65]. These up-regulated responses are orchestrated by senescence-associated hormones throughout the lifespan [65]. In cases of unexpected death, such as herbicides spraying on leaves, absorption primarily occurs through foliage instead of roots. Glyphosate application eventually leads to severe injuries, resulting in the senescence and death of the whole plant. Transcriptome analysis of the artificial death showed a greater number of DEGs in leaves compared to roots (Fig. 1), probably due to foliar absorption of glyphosate. The heightened responsiveness of leaf transcriptome aligned with the phenomenon of significant changes during senescence primarily targeting chloroplast degradation in leaves [22]. Moreover, such abrupt death disrupted the expression control, as 32–39% of expressed genes were sporadically distributed across samples (CV > 0.7), indicating unpredictable responses of the entire genome. In contrast, more genes exhibited stable expression during natural death (66–90% of genes displaying CV < 0.5) (Fig. 6A-F), reflecting a more regulated “willing to die” process. These findings suggested that acute death strongly impacts the physiology and expression pattern of healthy *Arabidopsis* plants.

In roots, the DEGs shared by RND2 and RAD were associated with three GO terms related to oxidative stresses (GO:0006979, GO:0055114 and GO:0042744) (Supplementary Table S14). The sharing pattern, along with the absence in natural senescence of leaves and RND1, suggested that the ability to resist oxidative pressure is crucial in facing life-threatening challenges, as contrast not revealed in the programmed senescence in leaves. This indicates that the natural death of leaves does not immediately impact plant survival, whereas in roots (both ND and AD), as well as the induced death in leaves, the accumulation of hydrogen peroxide or ROS may damage root tissues, leading to organ senescence/death [57]. ROS interacts with phytohormones, triggering chain reactions in organ senescence [41]. To delay life loss in both programmed and artificial deaths, antioxidant genes are thereby activated.

Despite the shared biological processes in leaves between natural and artificial deaths, the response of jasmonic acid (GO:0009753) was exclusively enriched in the DEGs of LND1, and oxidative stress response process (GO:0006979 response to oxidative stress and GO:0055114 oxidation-reduction process) occurred in LAD only (Fig. 3). We have proposed that *RRTF1* may transmit the JA signal to initiate root senescence. The early signal of root senescence was not activated following glyphosate treatment (Supplementary Table S13), suggesting a specific role of RRTF1 in regulating natural death of roots. Similarly, *PDF2.1* was only up-regulated during root death. Consequently, these pathways could not be induced by the ROS burst generated by glyphosate. Their roles were more likely associated with the alternation of generations, via well-controlled gene regulatory changes to secure nitrogen reallocation. In contrast, artificial death led to irregular fluctuation in gene expression (Fig. 6G, H). Besides, several macromolecule synthesis processes were identified exclusively in artificial death of roots, such as cellular amino acid biosynthetic process (GO:0008652) and flavonoid biosynthetic process (GO:0009813). Experiencing acute and irreversible death, *Arabidopsis* treated with glyphosate would struggle to recover, ultimately in vain. Taken together, although some death mechanisms of glyphosate-induced death were similar to those activated in natural death, certain genes managed the programmed death process to ensure the energy transfer to seeds for completing the last stage of life.

## Conclusion

**Fig. 7:**
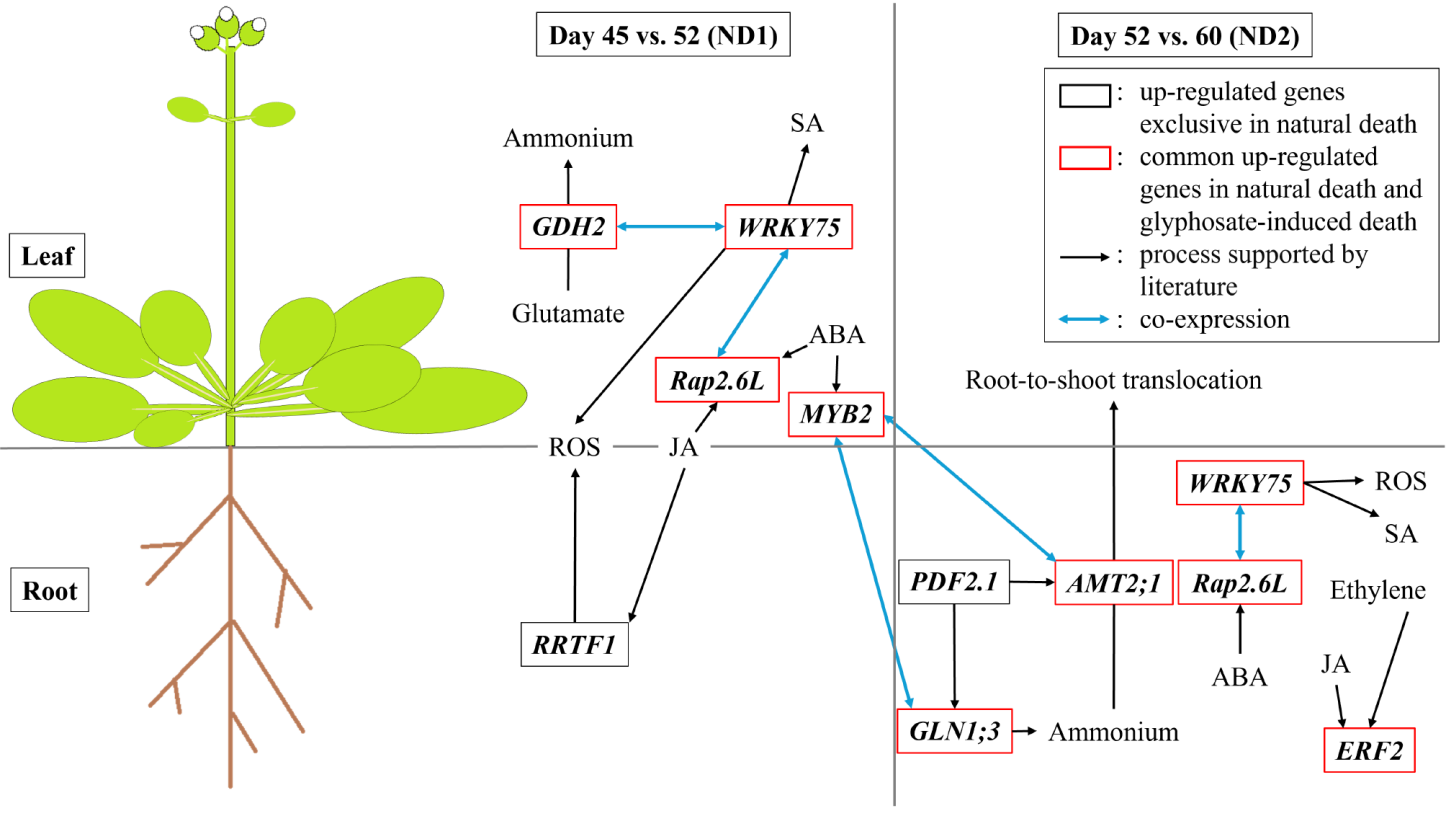
Regulation model of transcriptional gene-gene and gene-phytohormone interactions in leaves and roots during the whole-plant senescence in *Arabidopsis*. The phytohormone JA triggers early signaling of senescence. In leaves, JA, along with another phytohormone ABA, directly or indirectly induces the transcription factors *Rap2.6 L* and *WRKY75*, subsequently increasing *GDH2* expression to respond to local C/N imbalances caused by chloroplast degradation. On the other hand, RRTF1 and PDF2.1 transmit JA signals to initiate root senescence. In the later stages of senescence, PDF2.1 co-expressed with AMT2;1 and GLN1;3, which are involved in assimilation of ammonium and the translocation of ammonium from roots to shoots. These genes scavenge the ammonium released during root death and contribute to nutrient recycling in senescing roots

Senescence/death is a very complicated process, with genes of quite many pathways involved. Our result provides an overview of the relationship between phytohormones and ROS through *RRTF1*, *Rap2.6L*, *WRKY75*, and *PDF2.1* in natural death. *AMT2;1*, *GLN1;3*, and *GDH2* were involved in recycling and the root-to-shoot translocation of nitrogen (Fig. 7). From a reductionist perspective, the efficiency of energy transfer from parental organs to seeds ultimately dictates the fitness of *Arabidopsis* populations and their successors.

### Electronic supplementary material

Below is the link to the electronic supplementary material.

Supplementary Material 1

Supplementary Material 2

Supplementary Material 3

Supplementary Material 4

## Data Availability

The datasets generated and/or analysed during the current study are available in the NCBI SRA database under the Bioproject ID of PRJNA984411.

## Abbreviations

ABA: Abscisic acid
CV: Coefficient of variation
DEG: Differentially expressed gene
JA: Jasmonic acid
LAD: Leaf artificial death
LND: Leaf natural death
RAD: Root artificial death
RND: Root natural death
SA: Salicylic acid
