# Supplementary material for: Genome-wide expression analysis of vegetative organs during developmental and herbicide-induced whole plant senescence in Arabidopsis thaliana
Source: BMC Genomics. 2024 Jun 19;25:621. doi: 10.1186/s12864-024-10518-5 (PMC11188203; doi:10.1186/s12864-024-10518-5)
Supplement: Supplementary file 1 — Supplementary Material 1 [file 12864_2024_10518_MOESM1_ESM.docx]

**Supplementary data**

1. The phenotype of *Arabidopsis thaliana* in natural death.
2. The phenotype of *Arabidopsis thaliana* in glyphosate-treated death.
3. The results of RNA-seq.
4. The FPKM value for each sample.
5. The list of differentially expressed gene in each sample.
6. Biological process of down-regulated genes in LND1.
7. Biological process of down-regulated genes in LND2.
8. Biological process of down-regulated genes in LAD.
9. Biological process of down-regulated genes in RND1.
10. Biological process of down-regulated genes in RND2.
11. Biological process of down-regulated genes in RAD.
12. The list of JAZ-related genes.
13. The list of referred genes.
14. The list of oxidative stress-ralated genes shared with RND2 and RAD.
15. The fold change in expression of the marker genes Cab3, SAG12, and SEN4 at different leaf stages compared to day 39.

Table S1 The phenotype of *Arabidopsis thaliana* in natural death.

| Date | Description | Photo |
| --- | --- | --- |
| 0 day | Sowing |  |
| 2 days | Germination | 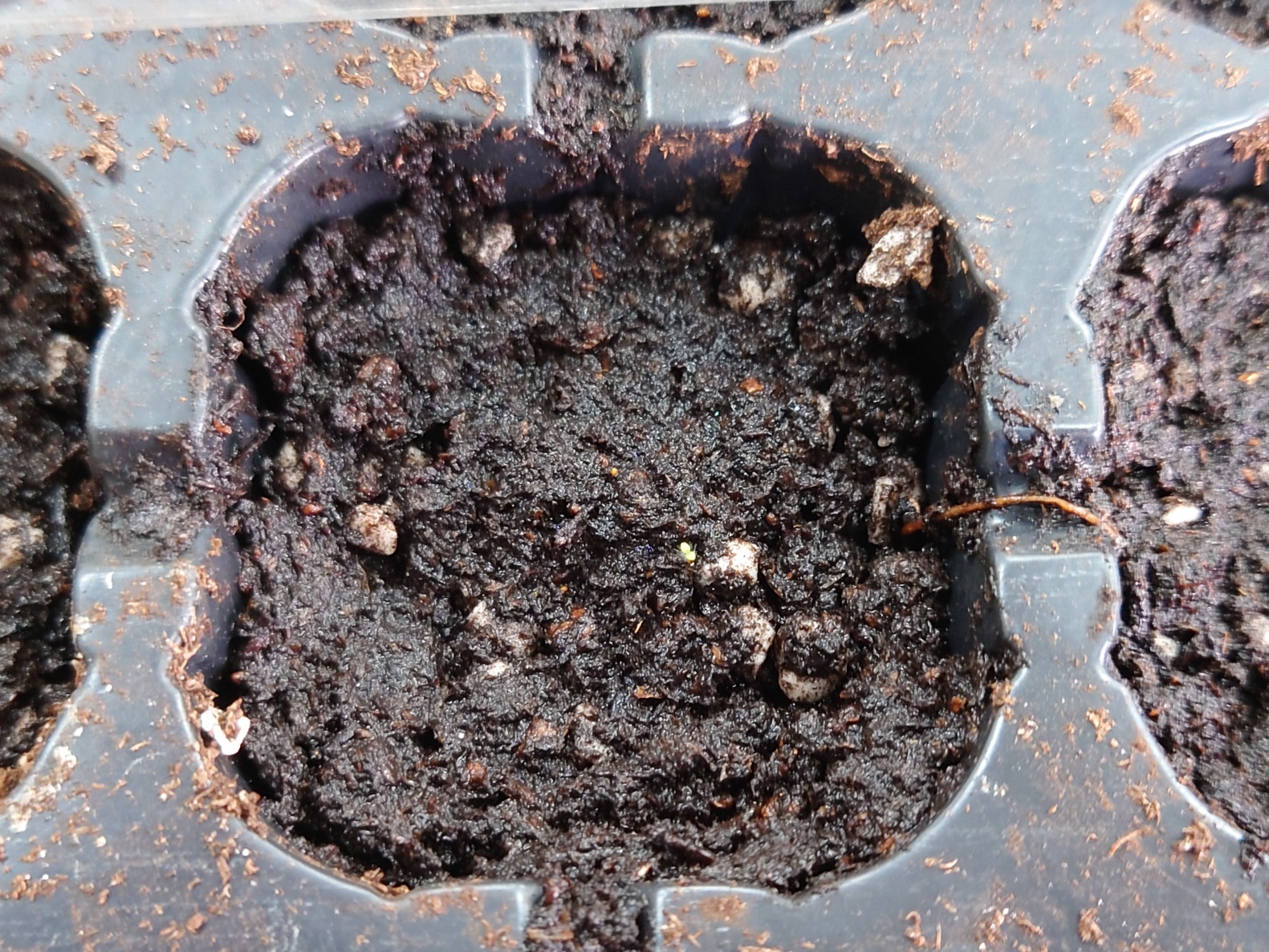 |
| 11 days | Two rosette leaves developing | 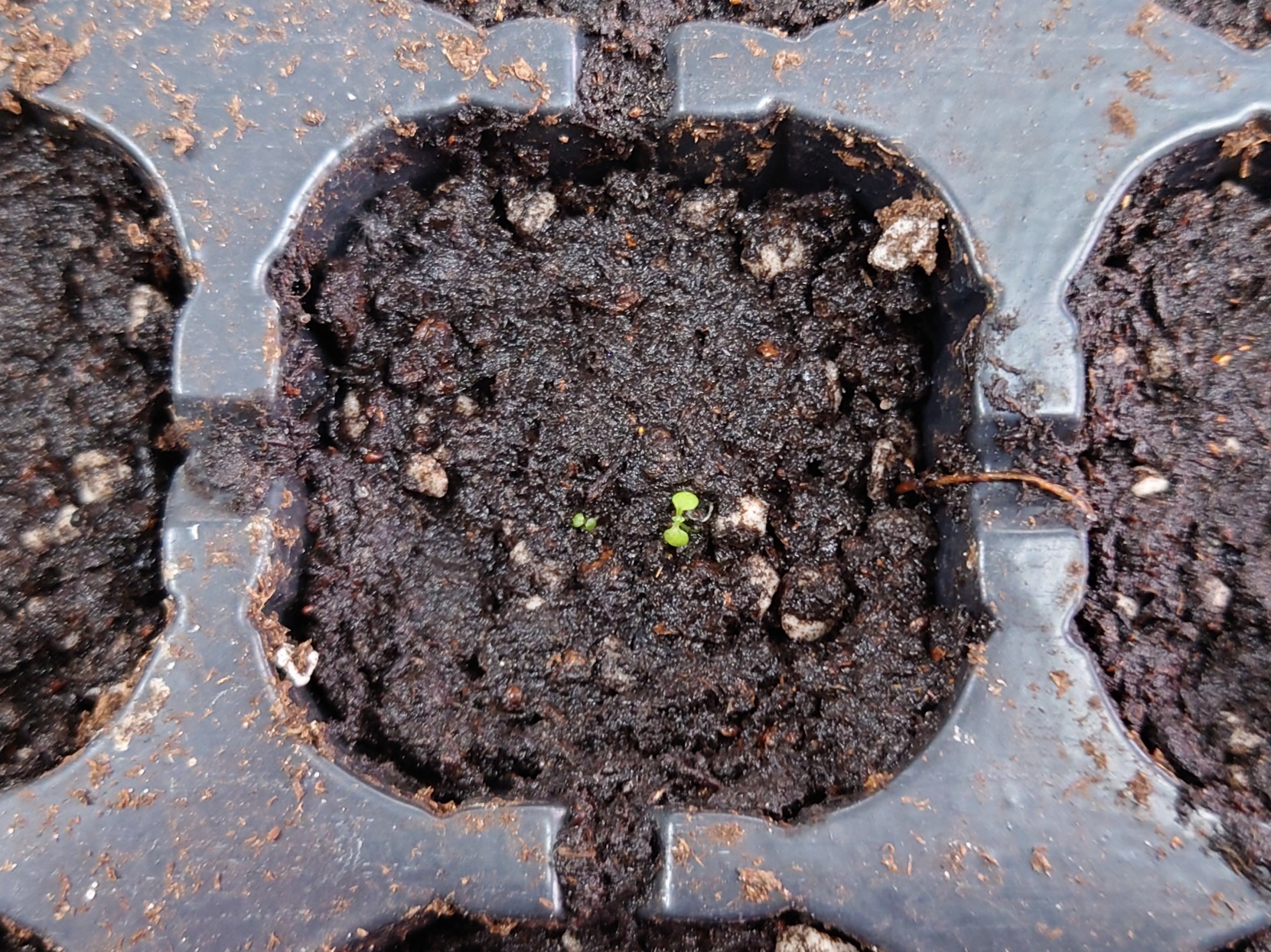 |
| 15 days | Four rosette leaves developing | 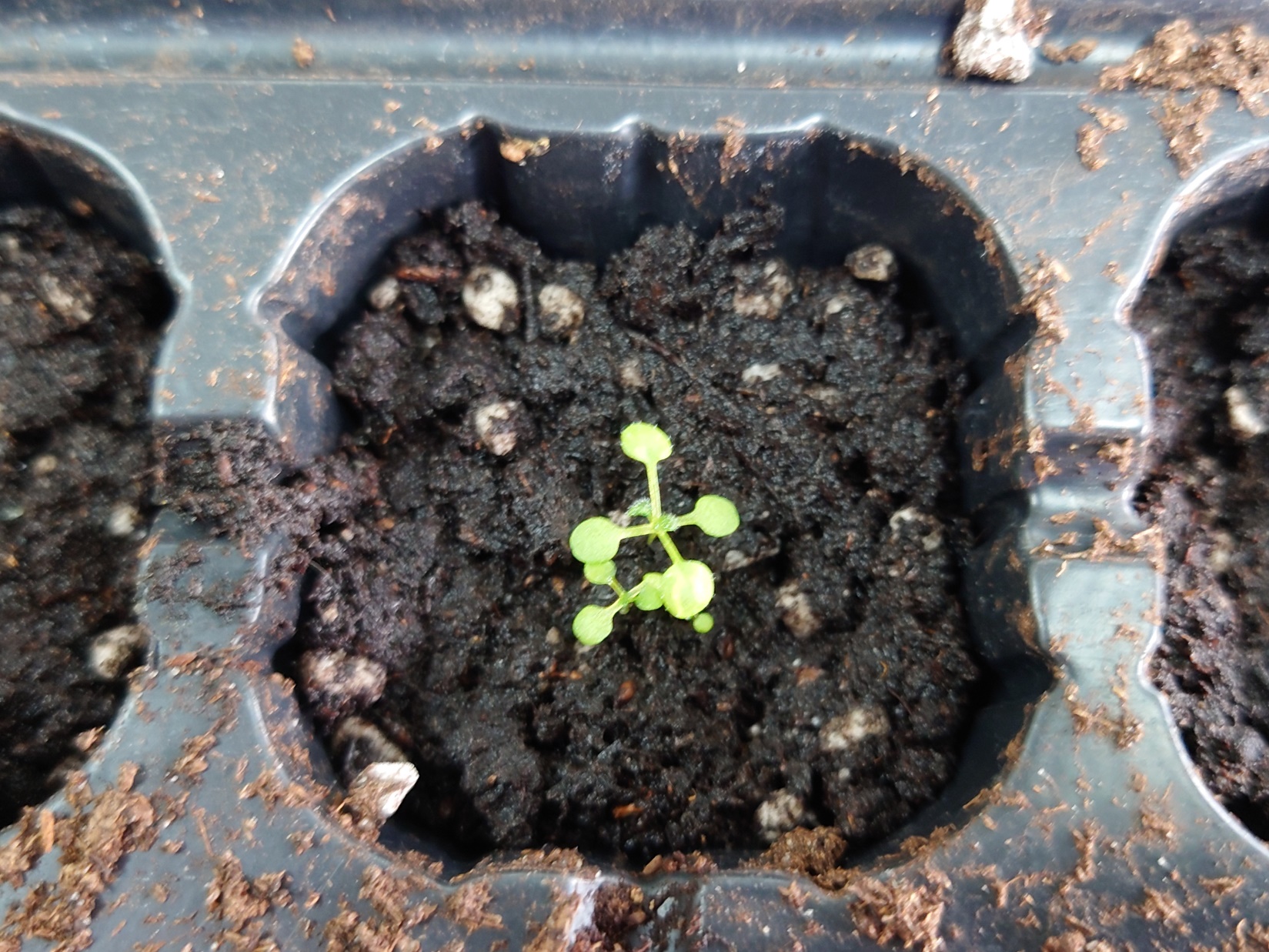 |
| 29 days | Fourteen rosette leaves developing | 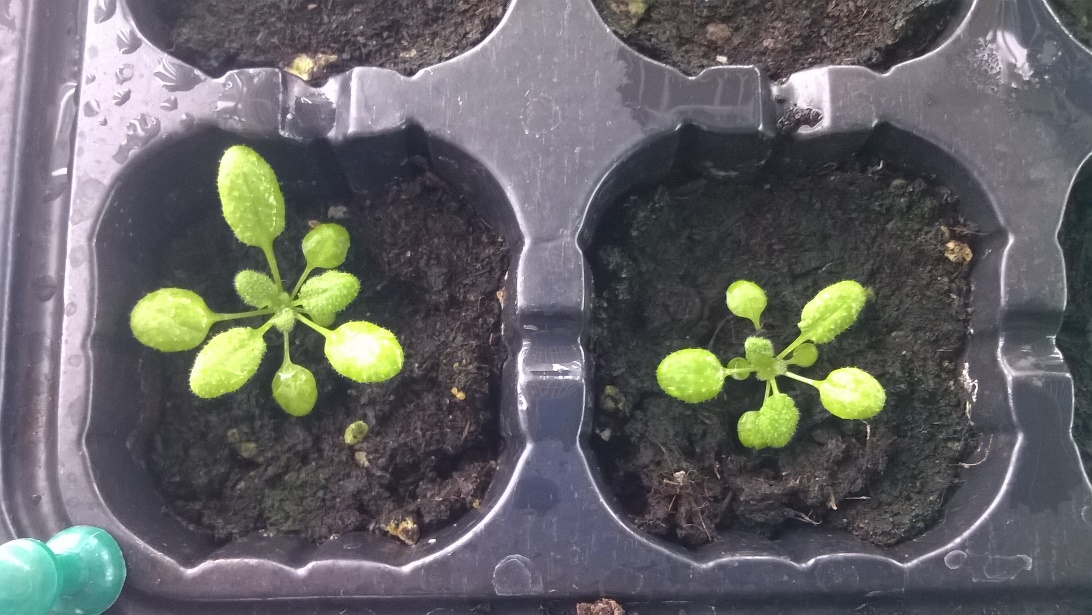 |
| 39 days | Start flowering | 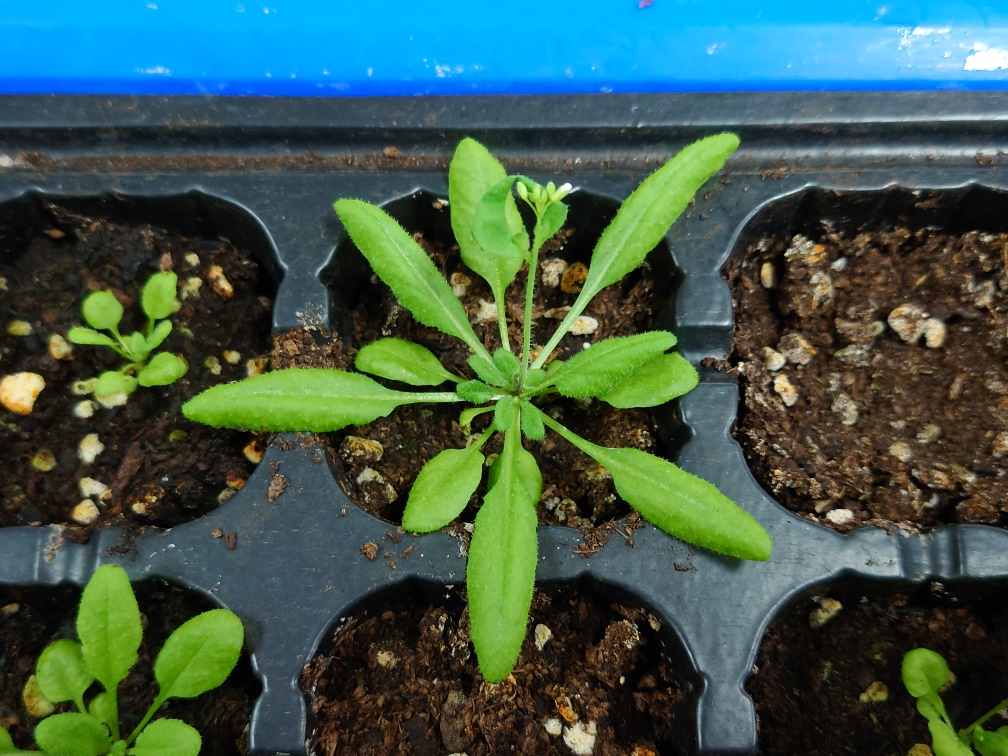 |
| 45 days | 50% flowering; rosette leaves start turning light green | 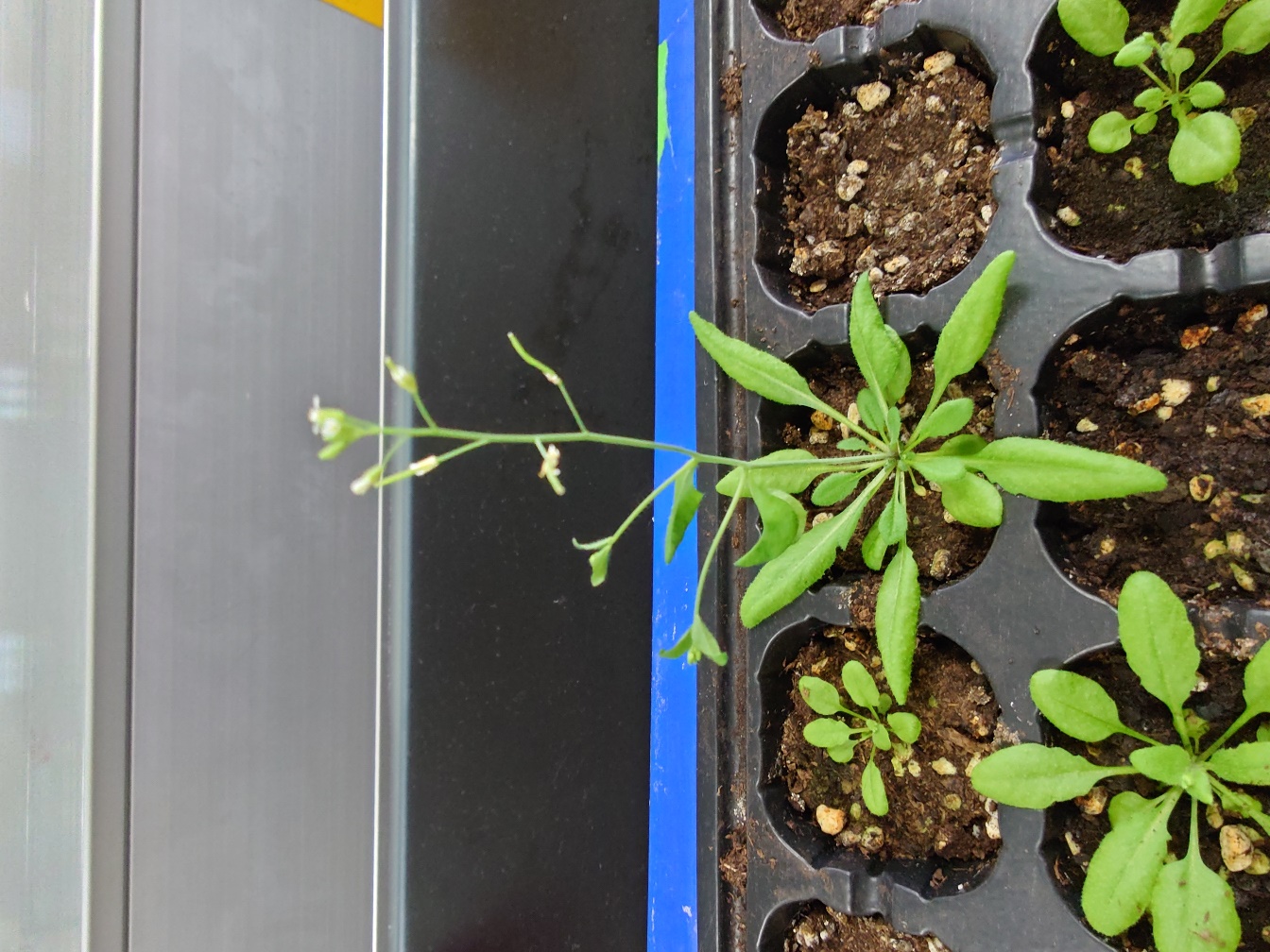 |
| 52 days | Siliques start maturing | 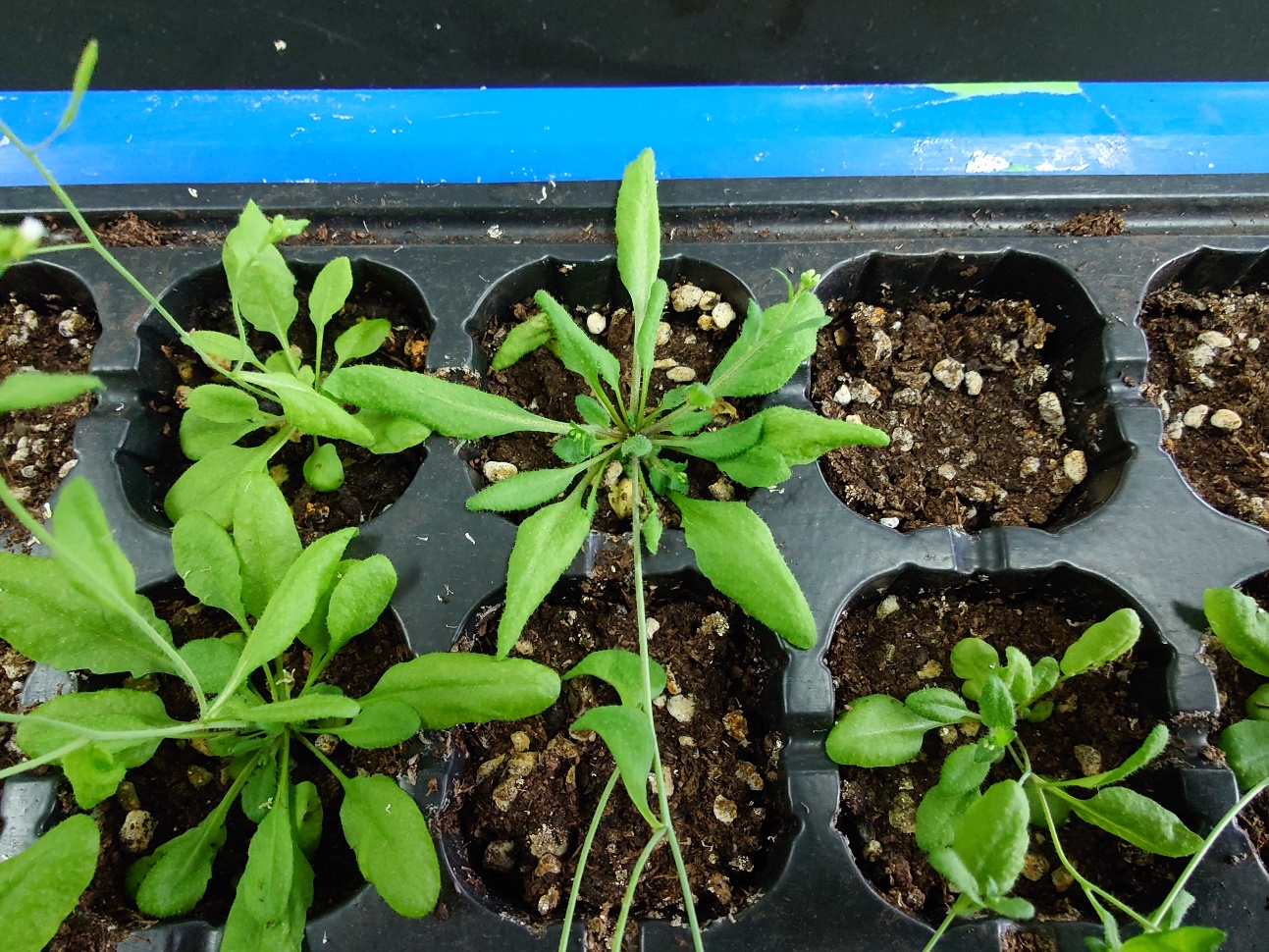 |
| 60 days | Rosette leaves start turning yellow | 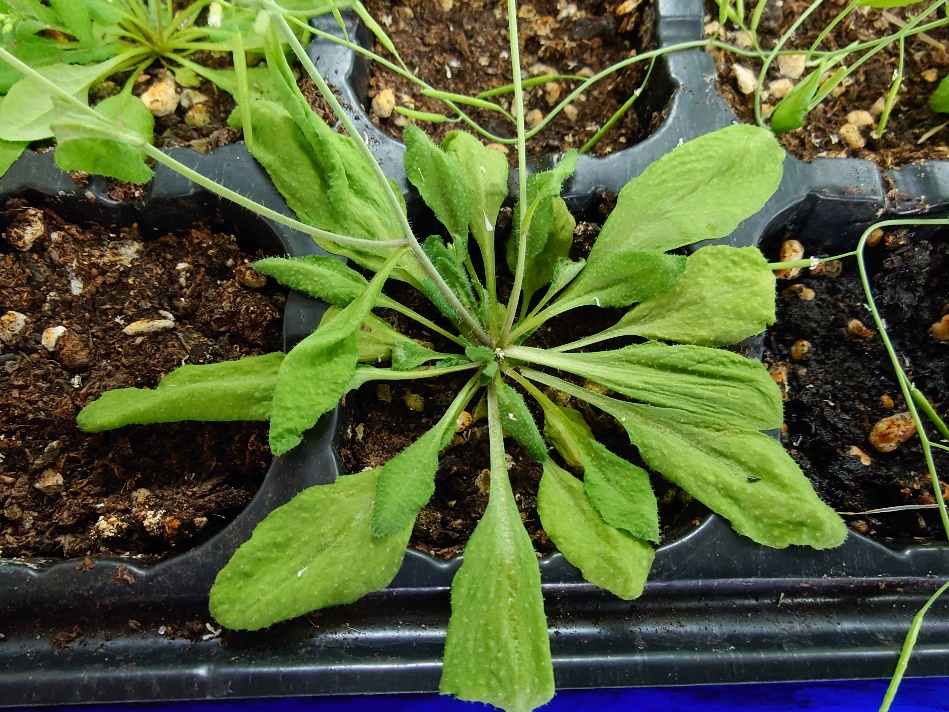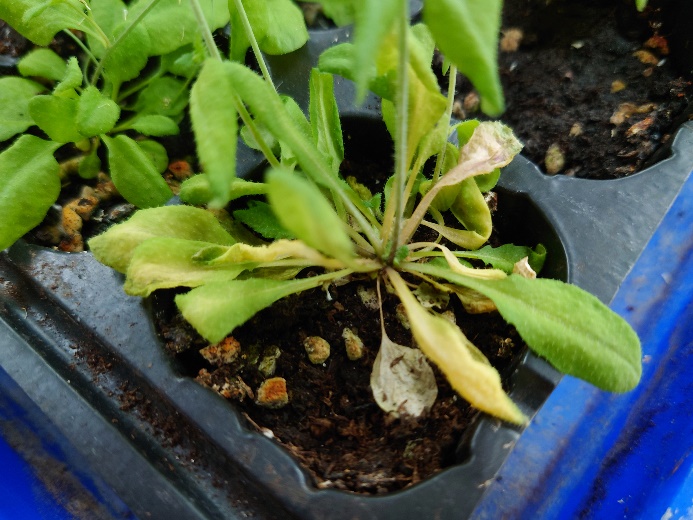 |

Table S2 The phenotype of *Arabidopsis thaliana* in glyphosate-treated death.

| Date | Description | Photo |
| --- | --- | --- |
| 0 day | Sowing |  |
| 3 days | Germination | 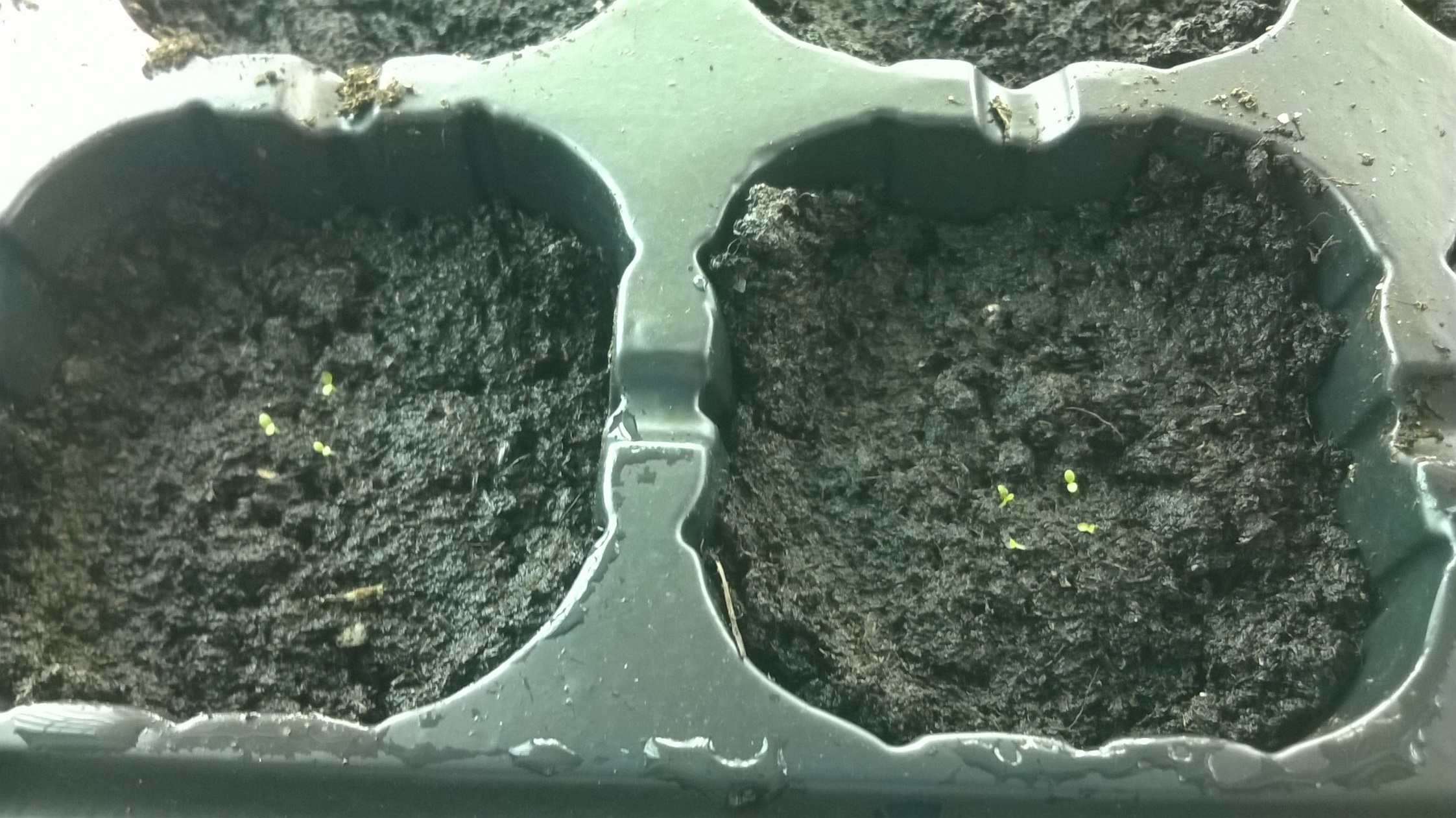 |
| 7 days | Two rosette leaves developing | 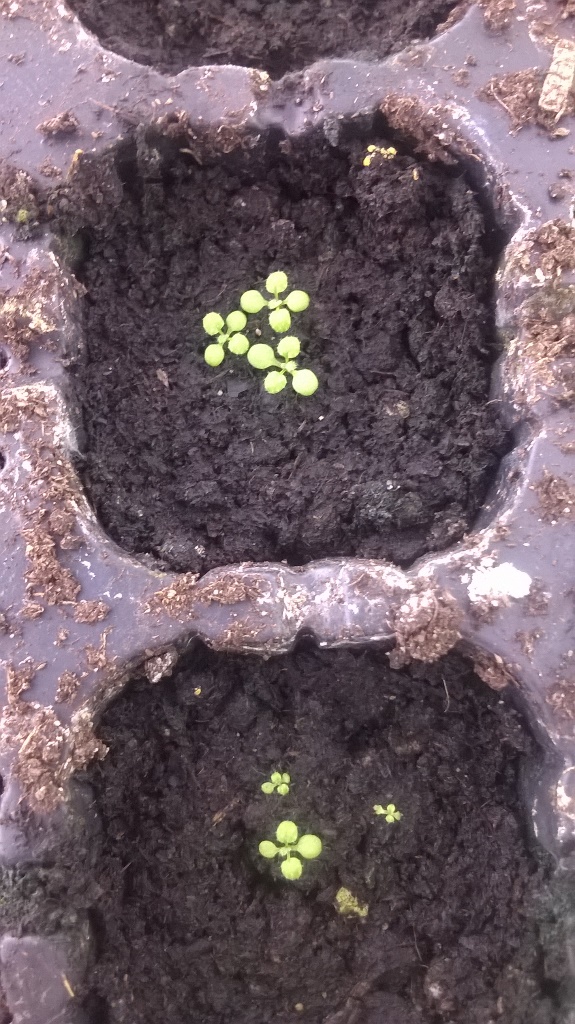 |
| 16 days | Four rosette leaves developing | 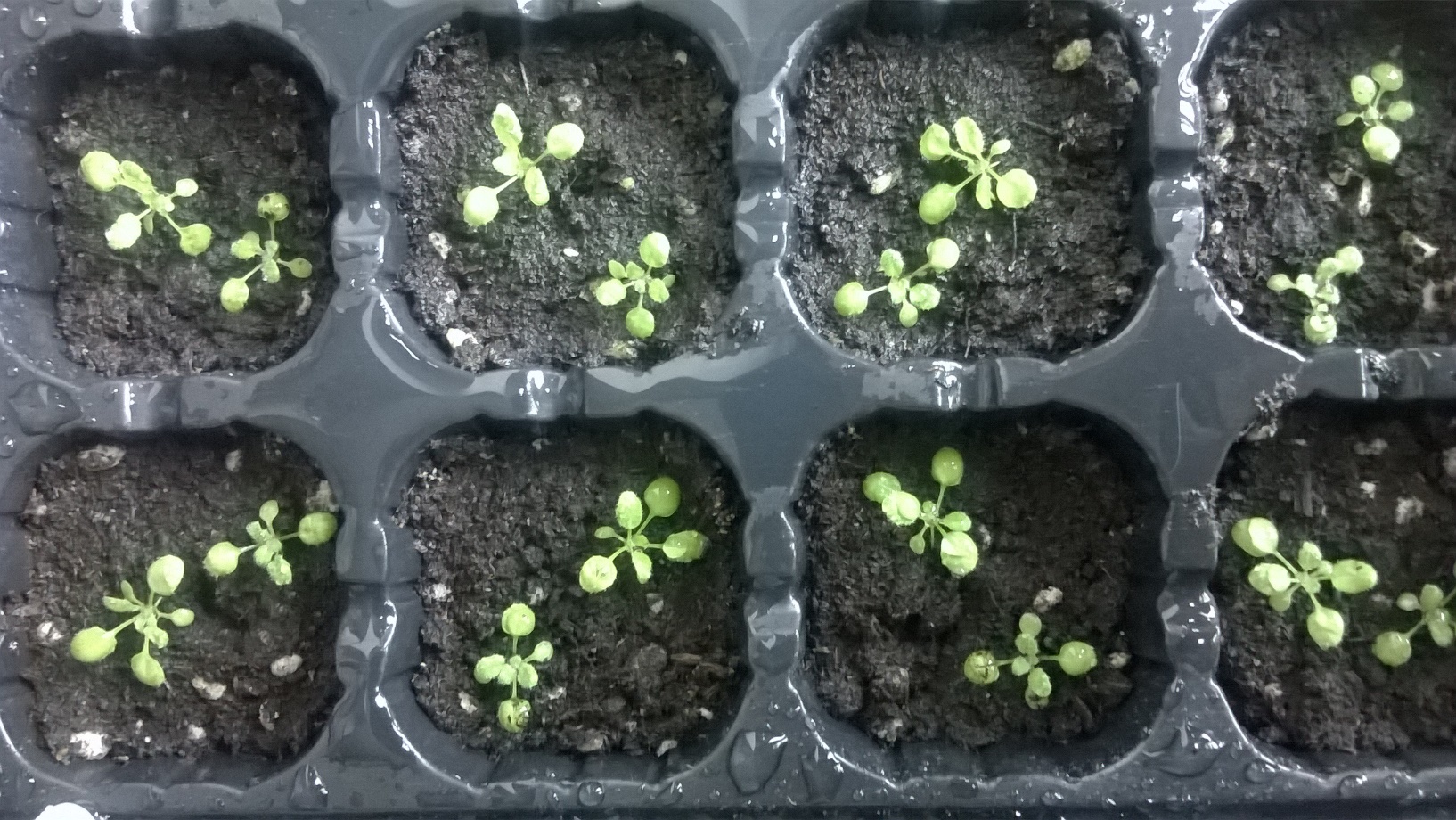 |
| 29 days | Fourteen rosette leaves developing | 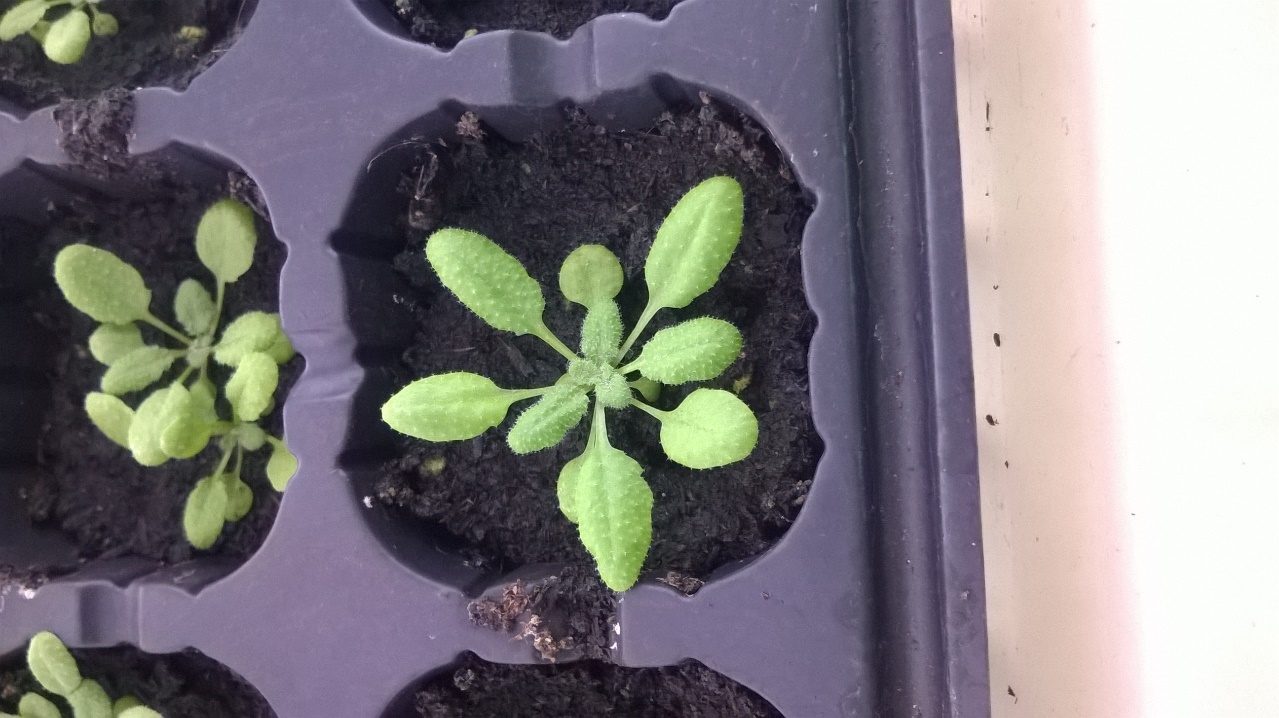 |
| 30 days | 1 DAT (day after treatment); rosette leaves turn yellow and become withered | 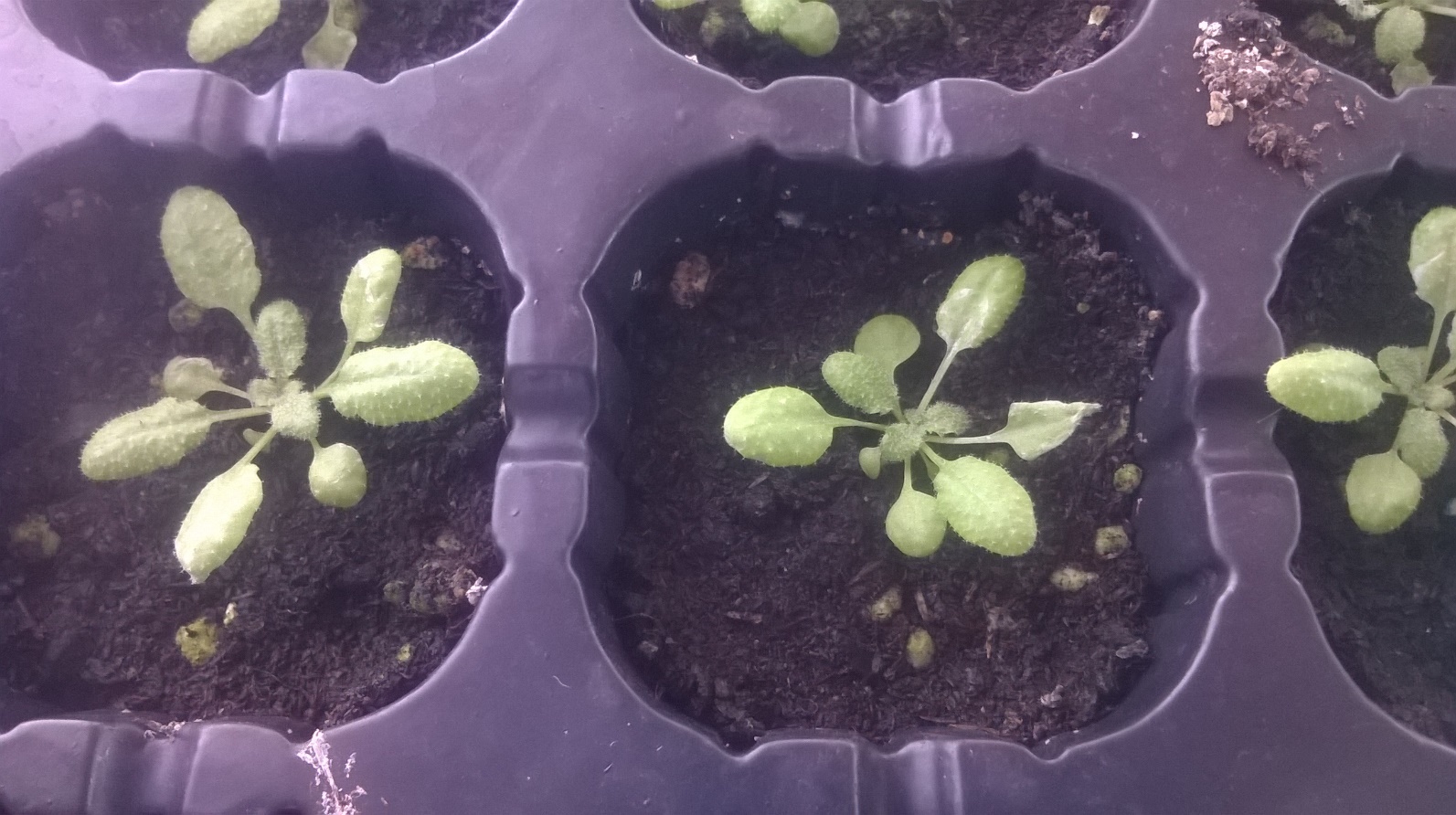 |
| 31 days | 2 DAT; rosette leaves shrivel | 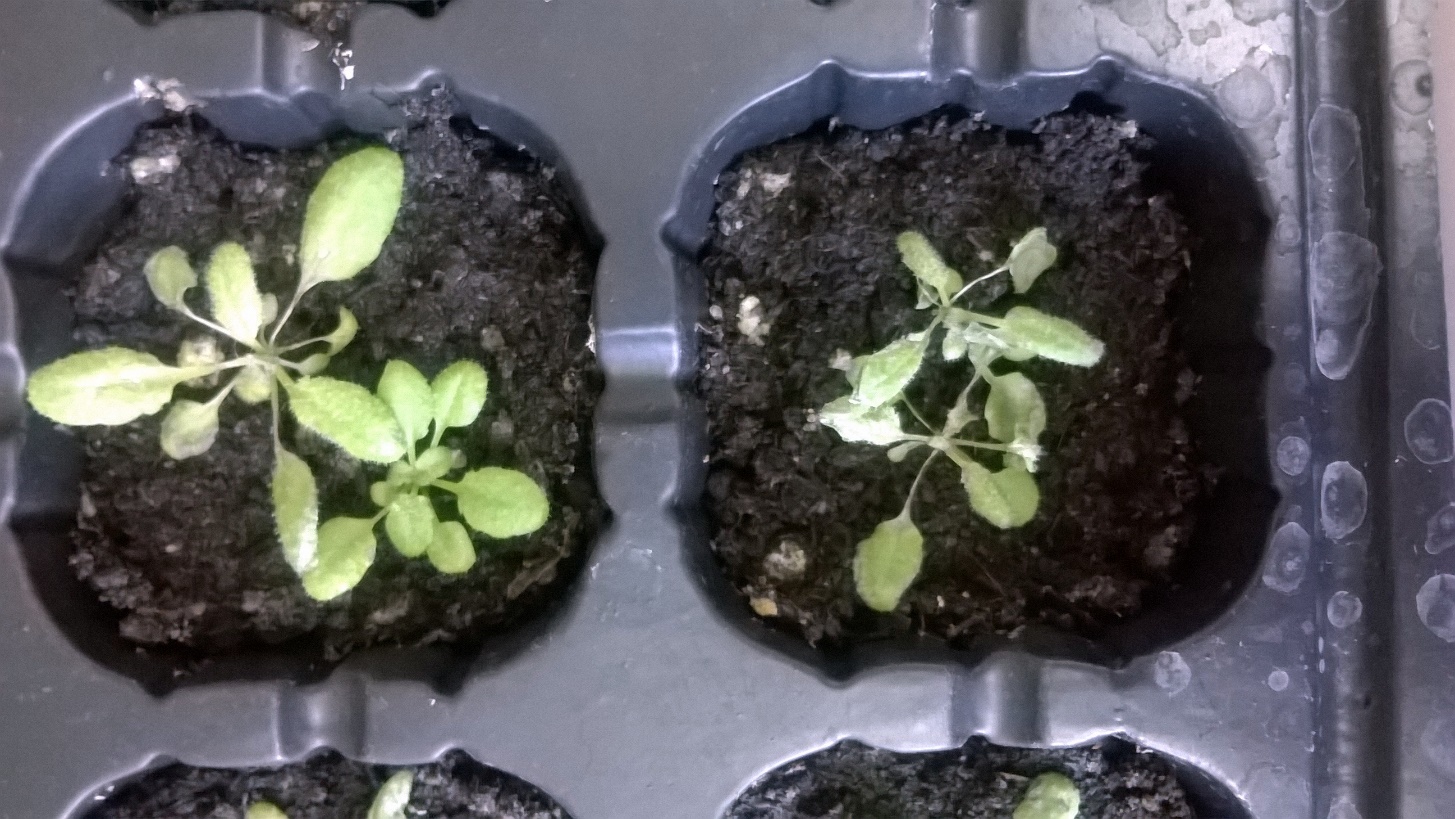 |

Table S3 The results of RNA-seq.

| **Sample** | **Total reads** | **Aligned paired reads** | **Alignment rate (%)** | **Detected genes** |
| --- | --- | --- | --- | --- |
| L_45 | 10,303,272 | 9,947,524 | 96.55 | 20,461 |
| L_52 | 11,807,435 | 11,305,985 | 95.75 | 20,355 |
| L_60 | 10,221,059 | 9,825,386 | 96.13 | 21,107 |
| R_45 | 11,047,700 | 10,435,808 | 94.46 | 21,908 |
| R_52 | 11,053,792 | 10,372,298 | 93.83 | 22,068 |
| R_60 | 11,992,068 | 10,824,633 | 90.26 | 21,604 |
| L_Gyphosate | 33,037,496 | 32,369,617 | 97.95 | 33,062 |
| L_Normal | 29,575,693 | 29,113,892 | 98.45 | 33,202 |
| R_Gyphosate | 36,323,088 | 35,612,101 | 98.05 | 33,019 |
| R_Normal | 34,795,172 | 33,940,510 | 97.55 | 33,267 |

Table S12 The list of JAZ-related genes.

| **Gene ID** | **Gene Name** | **LND1** | **LND2** | **RND1** | **RND2** | **LAD** | **RAD** | **Gene Description** |
| --- | --- | --- | --- | --- | --- | --- | --- | --- |
| AT1G15520 | ABCG40 | **+** | **nd** | **nd** | **+** | **+** | **+** | ATP-binding cassette G40 |
| AT5G42380 | CML37 | **+** | **nd** | **+** | **nd** | **+** | **+** | Calmodulin like 37 |
| AT1G17380 | JAZ5 | **+** | **nd** | **+** | **nd** | **+** | **+** | Jasmonate-zim domain protein 5 |
| AT2G34600 | JAZ7 | **+** | **nd** | **+** | **-** | **+** | **nd** | Jasmonate-zim domain protein 7 |
| AT1G30135 | JAZ8 | **+** | **nd** | **+** | **nd** | **+** | **+** | Jasmonate-zim domain protein 8 |
| AT5G13220 | JAZ10 | **+** | **nd** | **+** | **nd** | **+** | **+** | Jasmonate-zim domain protein 10 |
| AT1G72520 | LOX4 | **+** | **nd** | **+** | **nd** | **+** | **+** | Lipoxygenase 4 |
| AT4G34410 | RRTF1 | **nd** | **nd** | **+** | **nd** | **nd** | **nd** | Redox responsive transcription factor 1 |
| AT5G24780 | VSP1 | **+** | **-** | **nd** | **-** | **+** | **-** | Vegetative storage protein 1 |
| AT1G80840 | WRKY40 | **nd** | **nd** | **+** | **nd** | **+** | **+** | WRKY DNA-binding protein 40 |
| **+**: up-regulated; **-**: down-regulated; **nd**: no different | | | | | | | | |

Table S13 The list of referred genes.

| **Gene ID** | **Gene Name** | **LND1** | **LND2** | **RND1** | **RND2** | **LAD** | **RAD** | **Gene Description** |
| --- | --- | --- | --- | --- | --- | --- | --- | --- |
| AT2G38290 | AMT2;1 | **nd** | **nd** | **nd** | **+** | **+** | **nd** | Ammonium transporter 2 |
| AT5G47220 | ERF2 | **nd** | **nd** | **nd** | **+** | **+** | **+** | Ethylene responsive element binding factor 2 |
| AT5G07440 | GDH2 | **+** | **nd** | **nd** | **nd** | **+** | **nd** | Glutamate dehydrogenase 2 |
| AT3G17820 | GLN1;3 | **nd** | **nd** | **nd** | **+** | **+** | **+** | Glutamine synthetase 1;3 |
| AT2G34600 | JAZ7 | **+** | **nd** | **+** | **-** | **+** | **nd** | Jasmonate-zim domain protein 7 |
| AT5G13220 | JAZ10 | **+** | **nd** | **+** | **nd** | **+** | **+** | Jasmonate-zim domain protein 10 |
| AT1G72520 | LOX4 | **+** | **nd** | **+** | **nd** | **+** | **+** | PLAT/LH2 domain-containing lipoxygenase family protein |
| AT2G47190 | MYB2 | **+** | **nd** | **nd** | **nd** | **+** | **+** | MYB domain protein 2 |
| AT1G69490 | NAP | **nd** | **nd** | **nd** | **+** | **+** | **+** | NAC transcription factor gene family |
| AT1G71695 |  | **nd** | **nd** | **nd** | **+** | **-** | **+** | Peroxidase superfamily protein |
| AT2G37130 |  | **nd** | **nd** | **nd** | **+** | **+** | **+** | Peroxidase superfamily protein |
| AT2G02120 | PDF2.1 | **nd** | **nd** | **nd** | **+** | **nd** | **-** | Scorpion toxin-like knottin superfamily protein |
| AT4G34410 | RRTF1 | **nd** | **nd** | **+** | **nd** | **nd** | **nd** | Redox responsive transcription factor 1 |
| AT5G13330 | Rap2.6L | **+** | **nd** | **nd** | **+** | **+** | **+** | Related to AP2 6L |
| AT5G45890 | SAG12 | **nd** | **+** | **nd** | **nd** | **+** | **nd** | Senescence-Associated Gene 12 |
| AT2G29350 | SAG13 | **+** | **nd** | **nd** | **+** | **+** | **+** | Senescence-Associated Gene 13 |
| AT5G13080 | WRKY75 | **+** | **nd** | **nd** | **+** | **+** | **+** | WRKY DNA-binding protein 75 |
| **+**: up-regulated; **-**: down-regulated; **nd**: no different | | | | | | | | |

Table S14 The list of oxidative stress-related genes shared with RND2 and RAD.

| **Gene ID** | **Gene Name** | **Gene Description** |
| --- | --- | --- |
| AT5G59530 |  | 2-oxoglutarate (2OG) and Fe(II)-dependent oxygenase superfamily protein |
| AT1G08830 | CSD1 | Copper/zinc superoxide dismutase 1 |
| AT3G61280 | DUF821 | O-glucosyltransferase rumi-like protein |
| AT5G63030 | GRXC1 | Thioredoxin superfamily protein |
| AT1G53430 |  | Leucine-rich repeat transmembrane protein kinase |
| AT1G14870 | PCR2 | Plant cadmium resistance 2 |
| AT1G14540 | PER4 | Peroxidase superfamily protein |
| AT5G05340 | PRX52 | Peroxidase superfamily protein |
| AT3G49110 | PRXCA | Peroxidase CA |
| AT3G49120 | PRXCB | Peroxidase CB |
| AT1G49570 |  | Peroxidase superfamily protein |
| AT1G71695 |  | Peroxidase superfamily protein |
| AT2G37130 |  | Peroxidase superfamily protein |
| AT4G08780 |  | Peroxidase superfamily protein |
| AT4G11290 |  | Peroxidase superfamily protein |
| AT4G37520 |  | Peroxidase superfamily protein |
| AT5G06730 |  | Peroxidase superfamily protein |
| AT5G19880 |  | Peroxidase superfamily protein |
| AT5G58390 |  | Peroxidase superfamily protein |
| AT1G67000 |  | Protein kinase superfamily protein |
| AT1G51420 | SPP1 | Sucrose-phosphatase 1 |
| AT1G60740 |  | Thioredoxin superfamily protein |


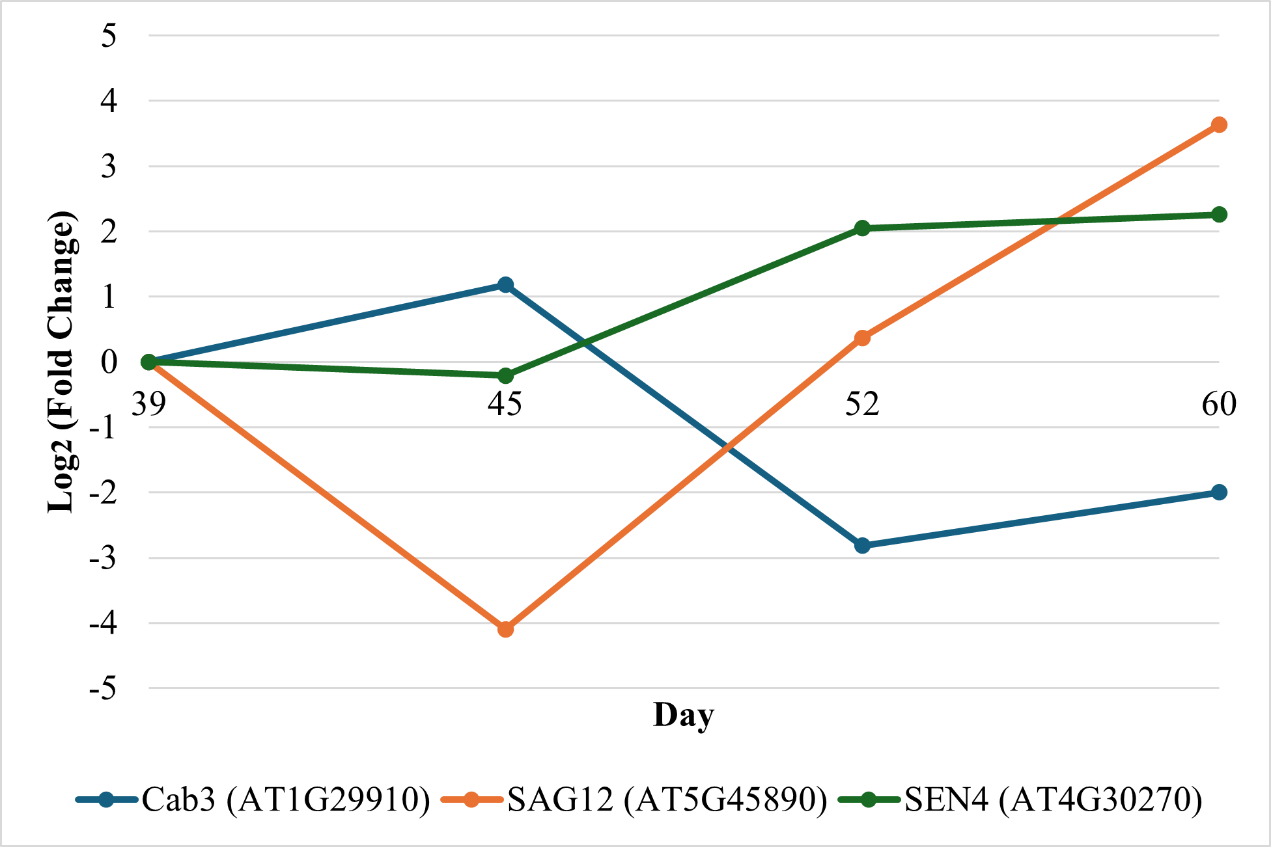


Figure S1 The fold change in expression of the marker genes Cab3, SAG12, and SEN4 at different leaf stages compared to day 39.
